# Preparation of an ion with the highest calculated proton affinity: *ortho*-diethynylbenzene dianion†
†Electronic supplementary information (ESI) available: Synthesis of precursor compounds, detailed experimental and theoretical procedures, 3 supporting figures, 4 supporting tables, NMR spectra of synthesised compounds. See DOI: 10.1039/c6sc01726f


**DOI:** 10.1039/c6sc01726f

**Published:** 2016-06-20

**Authors:** Berwyck L. J. Poad, Nicholas D. Reed, Christopher S. Hansen, Adam J. Trevitt, Stephen J. Blanksby, Emily G. Mackay, Michael S. Sherburn, Bun Chan, Leo Radom

**Affiliations:** a Central Analytical Research Facility , Institute for Future Environments , Queensland University of Technology , Brisbane , QLD 4001 , Australia . Email: berwyck.poad@qut.edu.au; b School of Chemistry , University of Wollongong , Gwynneville , NSW 2522 , Australia; c Research School of Chemistry , Australian National University , Canberra , ACT 2601 , Australia; d School of Chemistry , University of Sydney , Sydney , NSW 2006 , Australia

## Abstract

Owing to the increased proton affinity that results from additional negative charges, multiply-charged anions have been proposed as one route to prepare and access a range of new and powerful “superbases”. Paradoxically, while the additional electrons in polyanions increase basicity they serve to diminish the electron binding energy and thus, it had been thought, hinder experimental synthesis. We report the synthesis and isolation of the *ortho*-diethynylbenzene dianion (*ortho*-DEB^2–^) and present observations of this novel species undergoing gas-phase proton-abstraction reactions. Using a theoretical model based on Marcus–Hush theory, we attribute the stability of *ortho*-DEB^2–^ to the presence of a barrier that prevents spontaneous electron detachment. The proton affinity of 1843 kJ mol^–1^ calculated for this dianion superbase using high-level quantum chemistry calculations significantly exceeds that of the lithium monoxide anion, the most basic system previously prepared. The *ortho*-diethynylbenzene dianion is therefore the strongest base that has been experimentally observed to date.

## Abstract 2

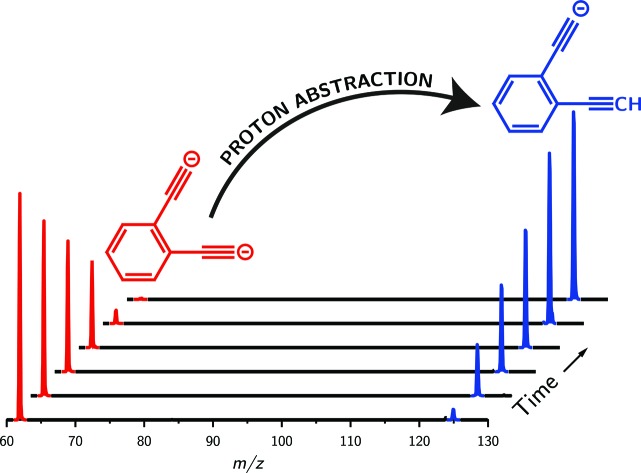
Owing to the increased proton affinity that results from additional negative charges, multiply-charged anions are shown as a route to preparing powerful ‘superbases’.

## Introduction

Exploration of the fundamental thermochemistry of acids and bases informs our understanding of chemical transformations and can drive innovation in the design of new reactions and reagents.^1^–^3^ The hydroxide anion has the largest proton affinity possible in an aqueous environment, since any base with a larger PA will abstract a proton from H_2_O (PA[OH^–^] = 1633.14 ± 0.04 kJ mol^–1^).^4^ To generate stronger bases in solution, non-aqueous solvents are required. For example lithium diisopropylamide, which is often employed in organic synthesis as a deprotonating agent, must be used in an aprotic solvent such as tetrahydrofuran.^5^ Such extremely strong bases are referred to as superbases.^6^ Owing to these environmental factors, investigation and comparison of the intrinsic basicity of compounds in solution is limited. Probing the reactivity of high proton affinity species in the gas phase, an environment free from any solvent interaction, provides an ideal way to investigate the fundamental basicity of these highly reactive systems.^7^

In the gas phase, the proton affinity of an anion is equivalent to the enthalpy of deprotonation (Δ_acid_*H*_298_) of the conjugate acid (*i.e.*, PA[X^–^] = Δ_acid_*H*_298_[XH]). The strongest base prepared to date is the lithium monoxide anion (LiO^–^).^8^ With an estimated proton affinity of 1782 ± 8 kJ mol^–1^, LiO^–^ supplanted the methide anion (CH_3_^–^) at the top of the basicity scale in 2008, exceeding the proton affinity of the carbanion by approximately 40 kJ mol^–1^.^4^,^9^,^10^ More recently, computational studies have proposed extending this framework to even more basic ions such as OLi_3_O^–^.^11^ However no clear synthetic route to form these ions in the gas phase has been demonstrated. Anionic superbases such as LiO^–^ and CH_3_^–^ necessarily satisfy two essential requirements: they are the conjugate bases of very weak gas-phase acids and their neutral radicals have low electron affinities (EAs). Multiply-charged anions can also fulfil these thermochemical requirements, as the gas-phase acidity of an anion is inherently low while the electron affinity of an anion (*i.e.*, the affinity for addition of a second electron to produce a dianion) can be low or even negative. Despite their potential instability, such dianion systems have been observed because of a repulsive Coulomb barrier (RCB) that arises from the interaction between the local bound-potential of the functional group carrying the charge (*e.g.*, a carboxylate group) and the repulsive Coulomb potential between like charges.^12^,^13^ This RCB can stabilise multiply-charged anions and, in some cases, allows for the generation and isolation of polyanions despite their negative electron binding energies.^14^,^15^ Based on these considerations, the 1,3-diethynylbenzene dianion (*meta*-DEB^2–^) was postulated to be a gas-phase superbase with a calculated PA of 1796.6 kJ mol^–1^, approximately 15 kJ mol^–1^ greater than that of the lithium monoxide anion.^8^ Thus far, no experimental studies have been reported on this gas-phase dianion – presumably due to the expectation that Coulomb repulsion between the proximate negative charges would destabilise the dianion and thus pose a challenge to its generation and isolation.

In this article, we outline the synthesis of *meta*-DEB^2–^ in the gas phase along with the isomeric 1,2- and 1,4-diethynylbenzene dianions (*ortho*- and *para*-DEB^2–^, respectively). Observation of proton-transfer reactions between these dianions and a number of weak acids demonstrates their behaviour as gas-phase bases. The calculated proton affinity of each of the DEB^2–^ isomers exceeds that of the lithium monoxide anion, with *ortho*-DEB^2–^ representing the strongest gas-phase base synthesised to date.

## Results and discussion

**Scheme 1:**
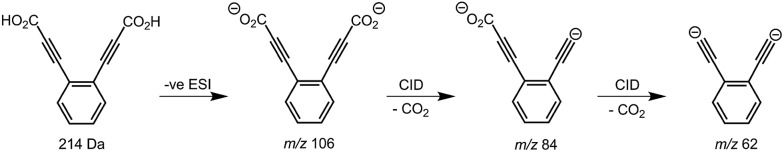
Gas-phase synthesis of the *ortho*-DEB isomer. Negative ion electrospray ionisation produces the dicarboxylate dianion (*m*/*z* 106). Subjecting this ion to successive stages of collisional activation results in the loss of two carbon dioxide molecules, with retention of both negative charges, yielding the *ortho*-DEB^2–^ dianion (*m*/*z* 62). Synthesis of the *meta*- and *para*-DEB^2–^ isomers proceeds in an analogous manner, using the appropriate diacid precursor.

**Fig. 1:**
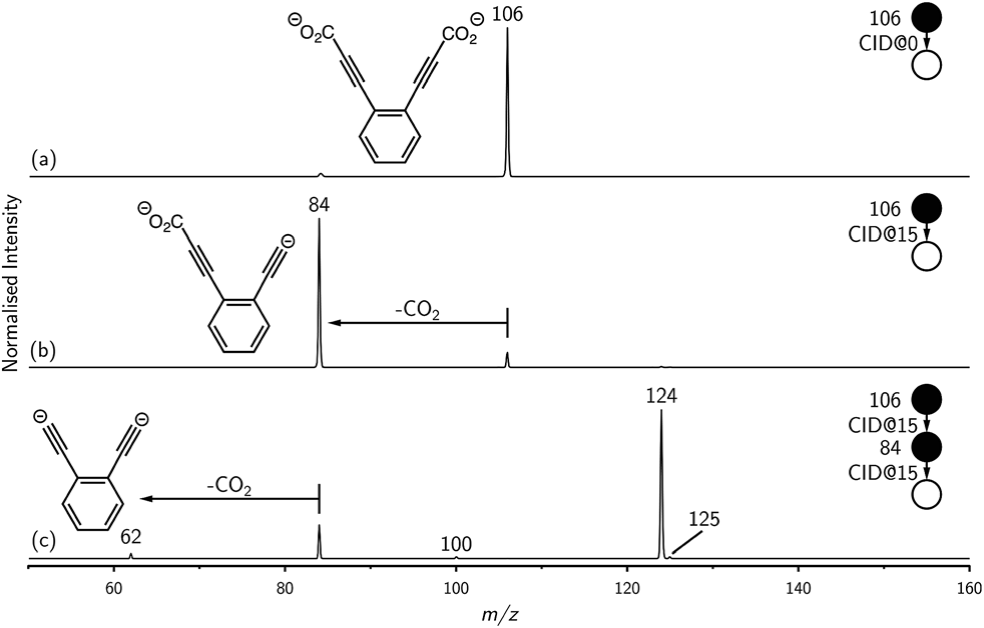
Mass spectra illustrating the synthesis of the *ortho*-DEB dianion base. The mass-isolated dicarboxylate anion at *m*/*z* 106 (a) is observed to decarboxylate under CID to yield *m*/*z* 84 (b). Subsequent isolation and activation of this *m*/*z* 84 ion yields a second decarboxylation product at *m*/*z* 62 and associated reaction products (c). The *meta*- and *para*-DEB dianions were synthesised using the same approach.

Synthesis of the *ortho*-DEB^2–^ dianion at a mass-to-charge ratio (*m*/*z*) of 62 was performed using tandem mass spectrometry in a linear quadrupole and followed the process outlined in Scheme 1 and Fig. 1. Negative ion electrospray ionisation of the diacid precursor generated the dicarboxylate dianion (*m*/*z* 106), which was mass-selected and subjected to successive collisional activation steps to remove the carboxylate groups while retaining both charges. Such decarboxylation processes that are accompanied by retention of charge have previously been noted for several organolithium compounds.^8^,^16^ The same method was deployed successfully for the generation of both *meta*- and *para*-DEB^2–^, using the appropriate isomeric diacid precursor. In addition to the DEB^2–^ dianion (*m*/*z* 62) and its associated proton-transfer product (*m*/*z* 125) observed in Fig. 1c, the main product ions following the final collisional activation step arise from loss of CO_2_ and loss of an electron from *m*/*z* 84 (*m*/*z* 124), accompanied by a small amount of C_2_ loss from this ion (*m*/*z* 100).

**Fig. 2:**
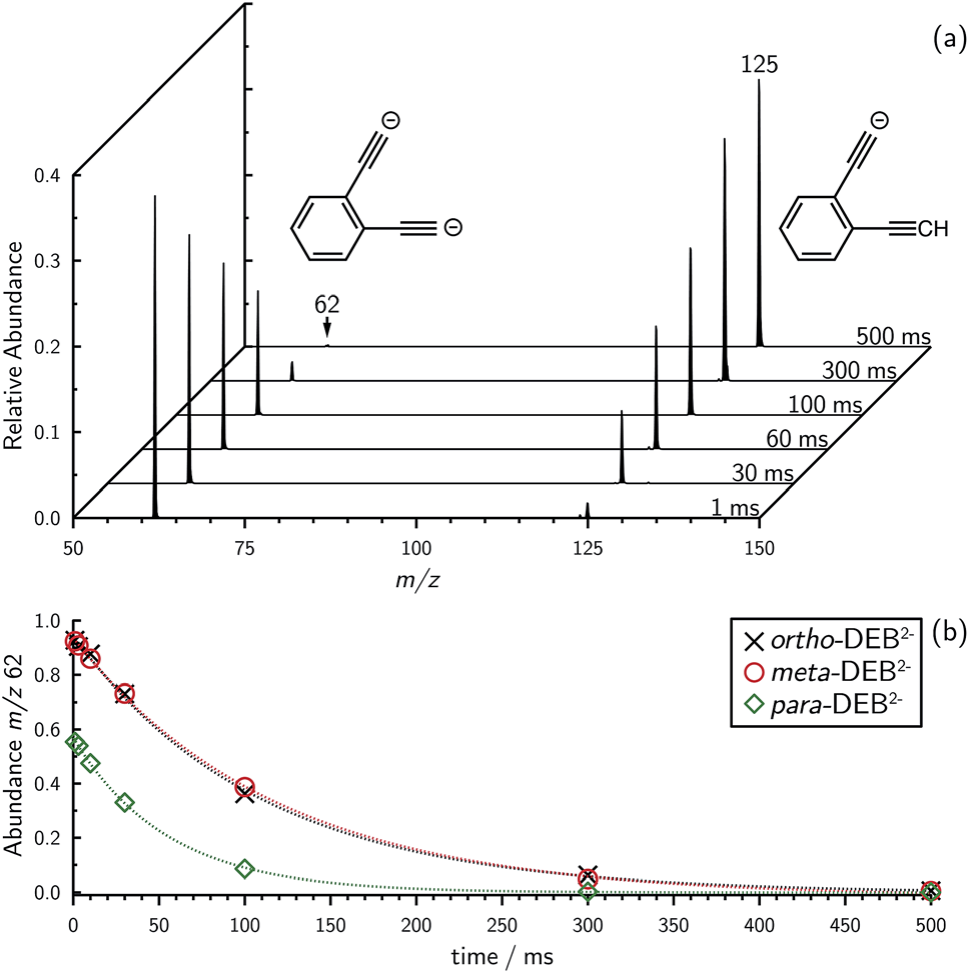
(a) Evidence for proton abstraction by *ortho*-DEB^2–^. Mass spectra acquired by isolating *ortho*-diethynylbenzene dianion (*m*/*z* 62) and monitoring the production of the proton-transfer product (*m*/*z* 125) for increasing trapping times in the presence of background water show that the ion signal intensity growth for the proton-transfer product is clearly coupled to the decay of the dianion superbase ion signal. (b) Decay plots showing the decrease in integrated ion signal intensity with increased trapping time for *m*/*z* 62 for all three DEB^2–^ dianions.

Decarboxylation of carboxylate anions upon collision-induced dissociation has previously been demonstrated as an effective means to prepare regiospecific anions in the gas phase.^17^ Such precedent strongly suggests that regiochemistry of the three *m*/*z* 62 dianions is retained from their precursor diacids (*i.e.*, *ortho*, *meta* and *para*-DEB^2–^). This is strongly supported by the differences in product ions and product ion abundances observed in the mass spectra at each step of the gas-phase preparation (*cf. ortho*-DEB^2–^ in Fig. 1 and *para*-DEB^2–^ in ESI Fig. S1†). Moreover, the pseudo first-order decay of each of the *m*/*z* 62 ion populations is consistent with only a single isomer in each instance (Fig. 2). Full experimental details, including preparation of the diacid precursors, are presented in the ESI.†

**Fig. 3:**
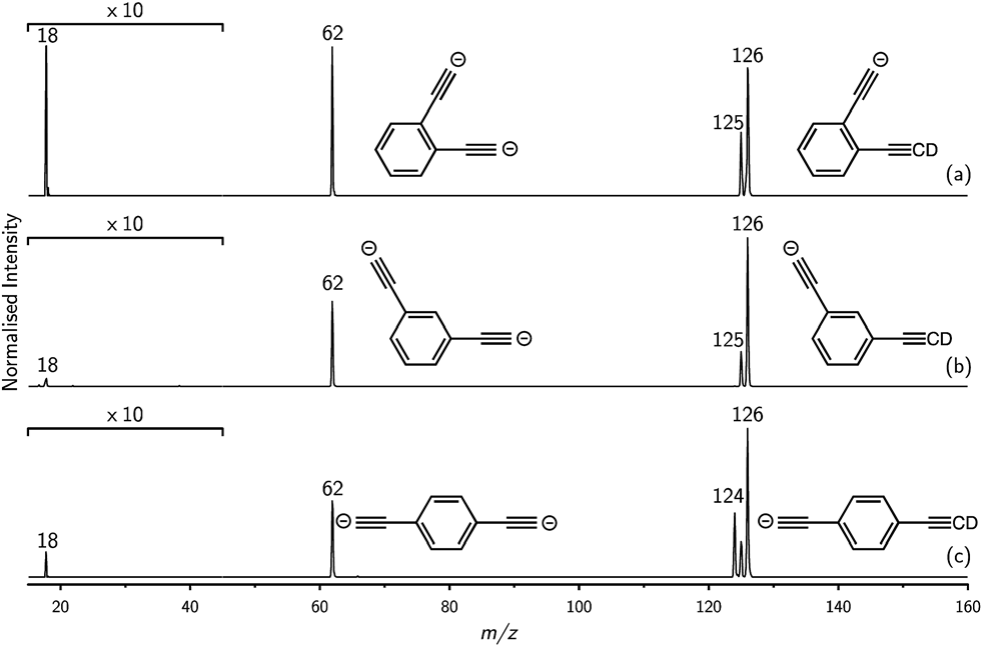
Comparison of the reactivity of (a) *ortho*-, (b) *meta*- and (c) *para*-DEB *m*/*z* 62 isomers towards D_2_O. The presence of *m*/*z* 18 (DO^–^) and *m*/*z* 126 (DEB^2–^ + D^+^) are indicative of deuteron abstraction from D_2_O. Note that the region from *m*/*z* 15–45 has been magnified by a factor of 10. Reaction time is 1 ms.

Mass selection allowed isolation of the *ortho*-DEB^2–^ dianion within the ion trap and an investigation of its fate over time. Interrogation of the contents of the ion trap at times ranging from 1–500 ms showed a decrease in the abundance of *ortho*-DEB^2–^ at *m*/*z* 62 and an associated increase in a product ion at *m*/*z* 125 (Fig. 2). This product arises from protonation of the dianion, most likely from background water present within the instrument. Proton transfer from water to the dianion was confirmed by leaking deuterated water into the ion trap. Mass spectra acquired following isolation of each of the three isomeric dianions and storage in the presence of D_2_O for 1 ms are displayed in Fig. 3. These spectra show the isotopic shift of the ion resulting from the acid–base reaction from *m*/*z* 125 to *m*/*z* 126, while the presence of *m*/*z* 18 (DO^–^) is diagnostic of deuteron abstraction from the heavy water. Because the performance of the ion-trap mass spectrometer is diminished at very low *m*/*z* (*i.e. m*/*z* < 20), the intensity of the *m*/*z* 18 peak appears artificially reduced compared with the intensity of *m*/*z* 126 and consequently a robust comparison of the product ion abundances is not possible. The presence of the *m*/*z* 125 ion in all spectra is evidence of proton abstraction from unlabelled water and other background gases present in the instrument.

For *ortho*- and *meta*-DEB^2–^ (Fig. 3a and b), proton or deuteron abstraction was the only process observed. For *para*-DEB^2–^ (Fig. 3c), a product ion arising from electron loss was also detected at *m*/*z* 124. This difference in the *para*-DEB^2–^ spectrum compared with that of the other two isomers can be rationalised by considering the calculated relative vertical detachment energies of the three dianions (*i.e.*, VDE[DEB^2–^]) and adiabatic electron affinities of the corresponding singly-charged radical anions (*i.e.*, AEA[DEB˙^–^]). Using a relation derived from Marcus–Hush theory, the RCB height can be estimated from the calculated AEA and VDE values according to eqn (1).^18^
1

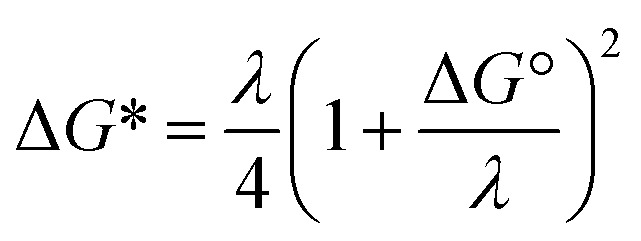




**Fig. 4:**
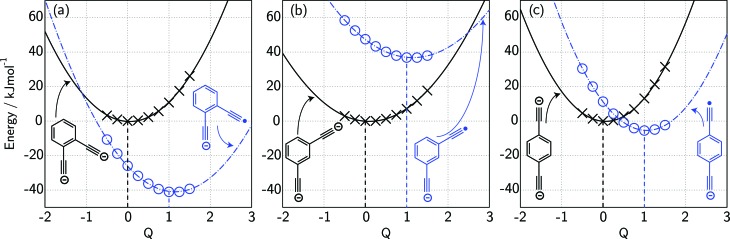
Electronic potential energy surfaces calculated at the G4(MP2)-6X//BMK/6-31+G(2df,p) level for the dianion (×) and monoanion radical (o) geometries for (a) *ortho*-DEB, (b) *meta*-DEB and (c) *para*-DEB. Geometries at various values of the parameter *Q* correspond to a linear interpolation/extrapolation between the minimum energy dianion geometry (*Q* = 0) and the minimum energy monoanion radical geometry (*Q* = 1).

If we interpret ΔG* as the energy barrier for electron detachment, the reorganisation energy *λ* as the energy difference between the VDE and AEA, and Δ*G*° as the AEA, an estimate of the RCB height can be ascertained from the computed minimum energy structures of diethynylbenzene as both singly- and doubly-charged anions. The results, compiled in Table 1, show a negative AEA for both *ortho*- and *para*-DEB˙^–^, suggesting that only the *meta*-DEB^2–^ dianion is thermodynamically stable with respect to electron detachment. For the three dianions, however, electron ejection is inhibited by RCBs of 11.1 (*ortho*), 52.7 (*meta*) and 1.9 (*para*) kJ mol^–1^. These barrier heights, calculated from eqn (1), are consistent with the analytical determination of barrier heights obtained using a rectilinear projection of the singly- and doubly-charged anion geometries (see Fig. 4 and ESI Table S1†). The presence of such barriers provides a rationalisation for the ability to prepare and isolate the DEB^2–^ dianions within the mass spectrometer despite the negative electron binding energies for two of the isomers. Furthermore, the low RCB barrier height for *para*-DEB^2–^ is consistent with the observed formation of the singly-charged *para*-DEB˙^–^ radical anion (*m*/*z* 124 in Fig. 3c) for this isomer, with the 1.9 kJ mol^–1^ barrier to electron loss able to be surmounted at the ∼310 K temperature in the ion trap.^19^

**Table 1:** Computed G4(MP2)-6X proton affinities (PA) at 298 K along with vertical detachment energies (VDE), adiabatic electron affinities (AEA) and Repulsive Coulomb Barrier (RCB) heights for the three isomeric diethynylbenzene (DEB) dianions. Corresponding values for LiO^–^, CH_3_^–^ and [C_2_–CH_2_–C_2_]^2–^ are provided for comparison

Species	PA/kJ mol^–1^	VDE/kJ mol^–1^	AEA^*a*^/kJ mol^–1^	RCB height/kJ mol^–1^
[*ortho*-DEB]^2–^	1843.3	–25.9	–41.0	11.1
[*meta*-DEB]^2–^	1786.8^*b*^	47.5	36.8	52.7
[*para*-DEB]^2–^	1780.7	11.3	–5.5	1.9
LiO^–^	1777.7^*c*^	50.8	50.1	—
CH_3_^–^	1747.7^*d*^	40.7	6.8	—
[C_2_–CH_2_–C_2_]^2–^	1888.1	–28.7	–77.1	4.2

^*a*^The AEA values refer to the radical species with one less negative charge, *e.g.* [*ortho*-DEB]˙^–^

^*b*^B3-LYP/6-311+G(2df,2pd) value of 1797 kJ mol^–1^.^8^

^*c*^Best theoretical estimate of 1782 ± 8 kJ mol^–1^.^8^

^*d*^Experimental values 1744 ± 12 kJ mol^–1^,^4^,^9^ 1742.2 ± 0.8 kJ mol^–1^.^10^

The demonstration that the DEB^2–^ isomers can deprotonate water indicates that they have a PA in excess of HO^–^, but does not quantify their strength. Just how strong are these dianion bases? Proton abstraction from weaker acids was investigated and ion–molecule reactions of DEB^2–^ dianions with benzene (ESI Fig. S2†) show the proton-transfer product at *m*/*z* 125 for all isomers along with the phenide ion at *m*/*z* 77 (C_6_H_5_^–^). These results provide experimental evidence that the DEB^2–^ isomers are capable of deprotonating benzene and thus have proton affinities in excess of PA[C_6_H_5_^–^] = 1678.7 ± 2.1 kJ mol^–1^.^4^ For both D_2_O and C_6_H_6_, the reactivity observed for *ortho*-DEB^2–^ far exceeds that of the other two isomers, reinforcing the heightened basicity of this species compared with *meta*- and *para*-DEB^2–^, which are essentially the same at this level of ion signal. No proton-transfer reactions were observed between DEB^2–^ isomers and either dihydrogen or methane despite favourable thermodynamics for these processes, which may be attributed to the presence of a substantial barrier for proton abstraction from these acids (ESI Fig. S3†).

The G4(MP2)-6X method has been shown to have good performance for computing PAs (±2.8 kJ mol^–1^) and benchmark calculations show good agreement in the case of strong gas-phase bases for which experimental PA data exist (Table S2†).^20^ Notwithstanding our experimental results, the computed G4(MP2)-6X proton affinities for all three DEB^2–^ dianions (Table 1) significantly exceed the experimentally benchmarked values for the methide anion (PA[CH_3_^–^] = 1747.7 kJ mol^–1^, see also Table S2†) as well as the proton affinity of lithium monoxide anion (PA[LiO^–^] = 1777.7 kJ mol^–1^) calculated at the same level of theory. Most significantly, *ortho*-DEB^2–^ has a computed proton affinity of 1843.3 kJ mol^–1^, making it the strongest base synthesised to date by some 65 kJ mol^–1^.

The thermochemical cycle of eqn (2) shows that the proton affinity of acetylide anions can be related to the homolytic bond dissociation enthalpy (BDE) of the C–H bond and the AEA of the corresponding radical by the ionisation energy of a hydrogen atom (IE[H]).^21^

2 PA[RC

<svg xmlns="http://www.w3.org/2000/svg" version="1.0" width="16.000000pt" height="16.000000pt" viewBox="0 0 16.000000 16.000000" preserveAspectRatio="xMidYMid meet"><metadata>
Created by potrace 1.16, written by Peter Selinger 2001-2019
</metadata><g transform="translate(1.000000,15.000000) scale(0.005147,-0.005147)" fill="currentColor" stroke="none"><path d="M0 1760 l0 -80 1360 0 1360 0 0 80 0 80 -1360 0 -1360 0 0 -80z M0 1280 l0 -80 1360 0 1360 0 0 80 0 80 -1360 0 -1360 0 0 -80z M0 800 l0 -80 1360 0 1360 0 0 80 0 80 -1360 0 -1360 0 0 -80z"/></g></svg>

C^–^] = BDE[RC

<svg xmlns="http://www.w3.org/2000/svg" version="1.0" width="16.000000pt" height="16.000000pt" viewBox="0 0 16.000000 16.000000" preserveAspectRatio="xMidYMid meet"><metadata>
Created by potrace 1.16, written by Peter Selinger 2001-2019
</metadata><g transform="translate(1.000000,15.000000) scale(0.005147,-0.005147)" fill="currentColor" stroke="none"><path d="M0 1760 l0 -80 1360 0 1360 0 0 80 0 80 -1360 0 -1360 0 0 -80z M0 1280 l0 -80 1360 0 1360 0 0 80 0 80 -1360 0 -1360 0 0 -80z M0 800 l0 -80 1360 0 1360 0 0 80 0 80 -1360 0 -1360 0 0 -80z"/></g></svg>

C–H] + IE[H] – AEA[RC

<svg xmlns="http://www.w3.org/2000/svg" version="1.0" width="16.000000pt" height="16.000000pt" viewBox="0 0 16.000000 16.000000" preserveAspectRatio="xMidYMid meet"><metadata>
Created by potrace 1.16, written by Peter Selinger 2001-2019
</metadata><g transform="translate(1.000000,15.000000) scale(0.005147,-0.005147)" fill="currentColor" stroke="none"><path d="M0 1760 l0 -80 1360 0 1360 0 0 80 0 80 -1360 0 -1360 0 0 -80z M0 1280 l0 -80 1360 0 1360 0 0 80 0 80 -1360 0 -1360 0 0 -80z M0 800 l0 -80 1360 0 1360 0 0 80 0 80 -1360 0 -1360 0 0 -80z"/></g></svg>

C˙]

It follows that the proton affinity of the diethynyl dianions is enhanced by the large, negative AEA of the corresponding radical anions. This effect of Coulombic repulsion on the AEA can be observed for the homologous series of model diethynyl dianions of the form [C=C–(CH_2_)_*n*_–C=C]^2–^ (*n* = 1–4) (see ESI Table S3†). As the charged moieties are progressively brought closer together, resulting in an increase in the Coulombic repulsion within the dianions, computed electron affinities for the [C=C–(CH_2_)_*n*_–C=C]˙^–^ radical anions decrease from +86.1 kJ mol^–1^ (*n* = 4) to +1.4 kJ mol^–1^ (*n* = 2) before returning large negative values when only a single methylene separates the two acetylide groups: –77.1 kJ mol^–1^ (*n* = 1). The decrease in AEA is accompanied by a concomitant increase in the proton affinity for the [C=C–(CH_2_)_*n*_–C=C]^2–^ dianion from 1775.4 (*n* = 4) through to 1888.1 kJ mol^–1^ (*n* = 1). While the proton affinity can be enhanced by bringing the like charges together, synthesis and isolation of a dianion superbase requires an RCB sufficient to prevent electron detachment. The simple formalism of eqn (1) predicts that when *n* = 1 the RCB is very low (4.2 kJ mol^–1^), suggesting that, like *para*-DEB^2–^ (RCB = 1.9 kJ mol^–1^, Table 1), the spontaneous loss of an electron is likely at room temperature.

## Conclusions

To prepare superbasic dianions, the lowered electron affinity of the multiply-charged anion must be balanced against the stabilising effect of the repulsive Coulomb barrier. The model proposed here indicates that it is possible to estimate the stability of a multiply-charged anion with respect to electron detachment, based solely on the computed energies of the dianion and the detached monoanion. Findings from this simple model system therefore suggest that the *ortho*-DEB^2–^ dianion occupies a somewhat privileged thermochemical position, possessing both a large, negative AEA and an RCB sufficient to prevent electron ejection. This suggests that this dianion superbase, with a proton affinity of 1843.3 kJ mol^–1^, may be difficult to displace from its position atop the gas-phase basicity scale.

## Experimental

### Synthetic methods

Full synthetic details, including NMR and mass spectra for all compounds synthesised, are provided in the ESI.† Briefly, the regioisomeric dicarboxylic acid precursors^22^ to the DEB anions were each prepared using a three-step sequence from the corresponding di-aldehyde. Thus, a two-fold Corey–Fuchs alkyne synthesis,^23^ involving firstly, Ramirez olefination^24^ to the bis-dibromoalkenes, then treatment with *n*-BuLi and *in situ* quenching of the double acetylide species with chloromethylformate gave the di-esters. Finally, ester hydrolysis gave the di-carboxylic acids.

### Mass spectrometry

The dianions were synthesised by electrospray ionisation of a methanolic solution of 3,3′-(phenylene)dipropiolic acid, basified with aqueous ammonia to aid deprotonation. Mass spectra were acquired using a dual linear quadrupole ion-trap mass spectrometer (LTQ Velos Pro, Thermo Scientific, San Jose CA). Precursor dianions at *m*/*z* 106 were isolated and collisionally activated (helium collision gas, 15% normalised collision energy^25^), yielding the first decarboxylation product at *m*/*z* 84. The singly-decarboxylated product was subsequently re-isolated and collisionally activated (15% normalised collision energy) to yield the doubly-decarboxylated dianion at *m*/*z* 62 along with the singly-charged CO_2_ loss ion *m*/*z* 124 and a proton-transfer product (*m*/*z* 125). This protocol was used for all three isomers. A minor product at *m*/*z* 100, most likely C_2_ loss from *m*/*z* 124, was observed for *ortho*-DEB^2–^ only.

Ion–molecule experiments were conducted by passing the ion-trap He buffer gas over a small amount of the neutral reagent (for D_2_O and C_6_H_6_). The vapour pressure of the neutral reagent at room temperature was sufficient to seed the helium buffer gas and was delivered to the high-pressure cell of the dual ion trap through the unmodified buffer gas inlet and split flow in the mass spectrometer. Reactions with gaseous reagents (D_2_ and CD_4_) were performed using a pre-made mixture of the deuterated reagents (both purchased from Sigma Aldrich, Castle Hill, NSW) in UHP helium (BOC Gases, North Ryde, NSW) and delivered into the ion trap through the ion trap buffer gas He inlet. The proportions of each gas mixture were 1.6% by volume in helium for D_2_ and 0.14% by volume in helium for CD_4_, yielding estimated number densities of 1.42 × 10^12^ molecules per cm^3^ and 1.24 × 10^11^ molecules per cm^3^, respectively, at the ∼2.5 mTorr pressure within the ion trap.^19^ The use of D_2_O, D_2_ and CD_4_ was necessitated by the presence of adventitious protonation reagents (such as H_2_O and CH_3_OH) in the vacuum system.

### Theoretical methods

Standard *ab initio* molecular orbital theory and density functional theory calculations were carried out with Gaussian09.^26^ Gas-phase geometries of stationary points were obtained with the BMK/6-31+G(2df,p) procedure.^27^ Following each geometry optimisation, harmonic frequency analysis was carried out to confirm the nature of the stationary point as an equilibrium structure (all real frequencies) or a transition structure (one imaginary frequency). To obtain the zero-point vibrational energies (ZPVEs) and thermal corrections for enthalpies at 298 K (Δ*H*_298_) for the fully-optimised structures, we used BMK/6-31+G(2df,p) harmonic vibrational frequencies and appropriate literature scale factors (0.9770 for ZPVE and 0.9627 for enthalpy correction).^20^,^28^ For each of *ortho*-, *meta*- and *para*-diethynylbenzene, we have examined the potential energy surfaces for the mono- and di-anionic species along the path that connects the geometries of the two ions. This is accomplished using structures obtained through a linear combination of the optimised structures for the mono- and di-anions (see Fig. 4). Improved single-point energies were evaluated using the G4(MP2)-6X procedure for all structures.^20^ All relative energies are given in kJ mol^–1^. Cartesian coordinates for all structures calculated are provided in the ESI as Table S4.†

## Supplementary Material

Supplementary information
