# Supplementary material for: Preparation of an ion with the highest calculated proton affinity: ortho-diethynylbenzene dianion
Source: Chem Sci. 2016 Jun 20;7(9):6245–50. doi: 10.1039/c6sc01726f (PMC6024202; doi:10.1039/c6sc01726f)
Supplement: Supplementary file 1 [file SC-007-C6SC01726F-s001.pdf]

Electronic Supplementary Material (ESI) for Chemical Science.
This journal is © The Royal Society of Chemistry 2016

Supporting Information for:

Preparation of an ion with the Highest Calculated Proton Affinity: Ortho-Diethynylbenzene dianion

Berwyck L. J. Poad,[1,2*] Nicholas D. Reed,[2] Christopher S. Hansen,[2] Adam J. Trevitt,[2] Stephen J. Blanksby,[1] Emily G. Mackay,[3] Michael S. Sherburn,[3] Bun Chan,[4,5] Leo Radom [4]

[1]Central Analytical Research Facility, Institute for Future Environments, Queensland University of Technology, Brisbane, QLD 4001, Australia.
[2]School of Chemistry, University of Wollongong, Wollongong, NSW 2522, Australia.
[3]Research School of Chemistry, Australian National University, Canberra, ACT 2601, Australia.
[4]School of Chemistry, University of Sydney, Sydney, NSW 2006, Australia.
[5] Present address: Graduate School of Engineering, Nagasaki University, Bunko 1-14, Nagasaki 852-8521, Japan

*Correspondence to: E-mail: berwyck.poad@qut.edu.au

**Synthetic Materials and Methods**	**Pages S2 – S11**
**Mass Spectrometry**	**Page S12**
**Theoretical Methods**	**Pages S12 – S13**
**Figures S1 – S3**	**Pages S14 – S15**
**Tables S1 – S4**	**Pages S16 – S22**
**^{1}H and ^{13}C NMR Spectra**	**Pages S23 – S28**
**Supporting References 1 – 24**	**Pages S29 – S30**

Synthetic Materials and Methods

Experimental procedures, reagents and glassware

Reactions were conducted in oven-dried glassware under a positive pressure of dry nitrogen. Solvents were dried using a method based upon that described by Grubbs and co-workers[1] or using standard laboratory procedures.[2] Commercially available chemicals were used as purchased, or purified by standard techniques.[2] Petroleum spirits refers to the fraction boiling at 30–40 °C. Solutions of *n*-BuLi were titrated before use according to the method of Lin and Paquette.[3]

NMR spectra

^{1}H NMR spectra were recorded at 400 MHz or 300 MHz using a Varian 400-MR or Varian Mercury 300 MHz spectrometer, as indicated. Residual solvent peaks were used as internal reference for ^{1}H NMR spectra ($CDCl_3$ δ 7.26 ppm). Coupling constants (J) are quoted to the nearest 0.1 Hz. Assignment of proton signals was assisted by COSY, HSQC and HMBC experiments. ^{13}C NMR spectra were recorded at 100 MHz using a Varian 400-MR spectrometer. Solvent peaks were used as internal reference for ^{13}C NMR spectra ($CDCl_3$ δ 77.16 ppm – central peak of the 1:1:1 triplet). Assignment of carbon signals was assisted by COSY, HSQC and HMBC experiments. The following abbreviations (or combinations thereof) are used to describe ^{1}H NMR multiplicities: s = singlet, d = doublet, t = triplet, m = multiplet.

IR spectra

IR spectra were recorded as KBr discs using a Perkin–Elmer 1600 FTIR spectrometer.

Mass spectrometry

Low resolution ESI mass spectra were recorded on a ZMD Micromass spectrometer. High resolution ESI mass spectra were recorded on a Waters LCT Premier time-of-flight (TOF) mass spectrometer.

Analytical TLC

Merck silica gel plates, pre-coated with silica gel 60 F254 (0.2 mm), were used for analytical TLC. Visualization was effected by quenching of UV fluorescence (λ_{max} = 254 nm) and by staining with *p*-anisaldehyde, potassium permanganate or phosphomolybdic acid TLC stain solution, followed by heating.

Flash chromatography

Merck Kiesegel 60 (230–400 mesh) silica gel was used for flash chromtography.

Melting points

Melting points are uncorrected, and were measured on a Reichert melting point apparatus.

1,2-Bis(2,2-dibromovinyl)benzene (2)

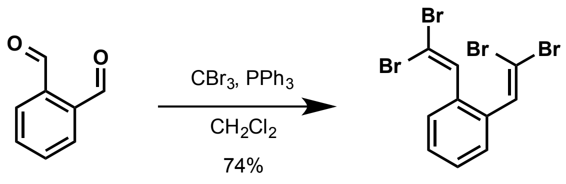

1 2

1,2-Bis(2,2-dibromovinyl)benzene **2** was prepared using a modified literature procedure.[4] Triphenylphosphine (22.5 g, 85.7 mmol, 4.6 mol. equiv.) was added portion-wise over 40 minutes to a solution of CBr_4 (14.2 g, 42.9 mmol, 2.3 mol. equiv.) in CH_2Cl_2 (200 mL) at 0 ºC under a nitrogen atmosphere. The reaction mixture was warmed to room temperature, and stirred for 1 h, before a solution of phthaldialdehyde (**1**) (2.50 g, 18.6 mmol, 1.0 mol. equiv.) in CH_2Cl_2 (80 mL) was added over 15 min. After stirring for 2 h at this temperature, H_2O (100 mL) was added. The layers were separated, and the aqueous layer was extracted with CH_2Cl_2 (3 × 60 mL). The combined organic layers were washed with brine (100 mL), dried over $MgSO_4$, filtered and concentrated under reduced pressure. The resulting solid was triturated three times with pentane, the combined pentane extracts were evaporated under reduced pressure and the resulting oil was purified *via* flash chromatography (SiO_2, petroleum spirits to 1% Et_2O/petroleum spirits) to give 1,2-bis(2,2-dibromovinyl)benzene **2** as a pale yellow solid (6.19 g, 74%).

Spectroscopic data matched those previously reported.[4]

R_f= 0.51 (5% EtOAc/petroleum spirits);

^{1}H NMR (400 MHz, $CDCl_3$): δ 7.52 (dd, J = 5.7, 3.4 Hz, 2H), 7.42 (s, 2H), 7.39 – 7.33 (m, 2H) ppm.

Dimethyl 3,3'-(1,2-phenylene)dipropiolic acid (3)

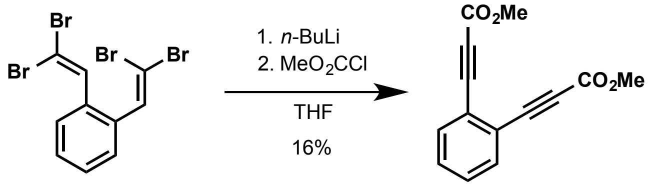

1,2-Benzene-bis(methyl propiolate) **3** was prepared by adopting and modifying the published procedure for the *meta*-isomer.[5] *n*-BuLi (11 mL, 1.6 M in hexanes, 18 mmol, 4.0 mol. equiv.) was added dropwise over 20 minutes to a solution of tetrabromide **2** (2.0 g, 4.5 mmol, 1.0 mol. equiv.) in THF (150 mL) at 0 ºC under a nitrogen atmosphere. The reaction mixture was stirred at this temperature for 30 min, before being transferred by cannula into a solution of methyl chloroformate (2.8 mL, 36 mmol, 8.0 mol. equiv.) in THF (15 mL) at 0 ºC. The reaction mixture was allowed to warm to room temperature over 30 min, before sat. aq. NH_4Cl (25 mL) was added. The aqueous layer was extracted with CH_2Cl_2 (3 × 20 mL), dried with $MgSO_4$, filtered and concentrated under reduced pressure. The crude product was then purified *via* flash chromatography (SiO_2, petroleum spirit to 30% EtOAc/petroleum spirits) to give 1,2-benzene-bis(methyl propiolate) **3** as a pale orange solid (0.17 g, 16%).

R_f = 0.44 (30% EtOAc/petroleum spirits);

m.p. = 80.8-82.3 °C (EtOAc/hexanes);

^{1}H NMR (400 MHz; $CDCl_3$): δ 7.62 (dd, J = 3.4, 2.3 Hz, 2H), 7.45 (dd, J = 3.6, 2.3 Hz, 2H), 3.86 (s, 6H) ppm;

^{13}C NMR (100 MHz; $CDCl_3$): δ 154.1 (C_q), 133.5 (CH), 130.3 (CH), 123.5 (C_q), 84.4 (C_q), 83.1 (C_q), 53.0 (CH_3) ppm;

IR (KBr disc): 3005, 2956, 2225, 1707 cm^{-1};

HRMS (ESI$^+$): calculated for $[C_{14}H_{10}O_4K]^+$: 281.0216; found 282.0214.

3,3'-(1,2-Phenylene)dipropiolate (4)

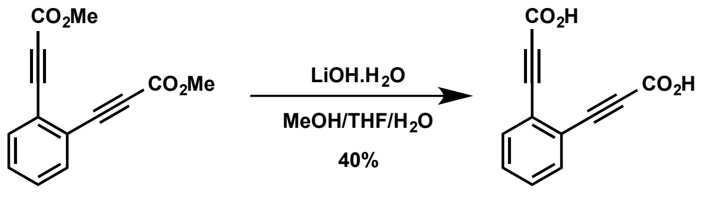

3 4

1,2-Benzenedipropynoic acid **4** was prepared by adopting and modifying a reported procedure on a structurally related compound.[6] LiOH.H_2O (37 mg, 0.89 mmol, 2.50 mol. equiv.) was added to a solution of diester **3** (86 mg, 0.36 mmol, 1.00 equiv.) in 6 mL MeOH/THF/H_2O (4:1:1) at room temperature. After stirring at this temp for 20 h, the reaction mixture was acidified (until pH ≤ 2) *via* the dropwise addition of conc. HCl, then extracted with CH_2Cl_2, dried ($MgSO_4$), filtered and concentrated under reduced pressure. The compound was then crystallised from CH_2Cl_2 to give 1,2-benzenedipropynoic acid **4** as white powder (30 mg, 40%).

Spectroscopic data matched those previously reported.[7]

^{1}H NMR (400 MHz, CD_3CN): δ 7.71 (dd, J = 5.8, 3.3 Hz, 2H), 7.57 (dd, J = 5.8, 3.3 Hz, 2H) ppm.

1,3-Bis(2,2-dibromovinyl)benzene (7)

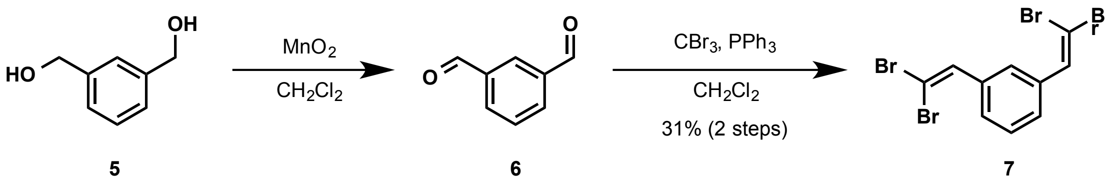

5 6 7

Bis-aldehyde **6** was prepared using a modified literature procedure.[8] Diol **5** (5.0 g, 36.2 mmol, 1.00 mol. equiv.) was added to a stirred suspension of MnO_2 (31.5 g, 362 mmol, 10.0 mol. equiv.) in CH_2Cl_2 (300 mL) at reflux under a nitrogen atmosphere. The reaction

mixture was held at reflux for a further 16 h, before being cooled to room temperature and filtered through Celite. Evaporation of the solvent under reduced pressure gave the crude bis-aldehyde **6** (5.0 g) as a colourless oil, which was used in the next reaction without further purification.

1,3-Bis(2,2-dibromovinyl)benzene **7** was prepared using a literature procedure.[4] Triphenylphosphine (43.6 g, 166 mmol, 4.6 mol. equiv.) was added portionwise over 1 h to a solution of carbon tetrabromide (27.6 g, 83.2 mmol, 2.30 mol. equiv.) in CH_2Cl_2 (250 mL) at 0 ºC under a nitrogen atmosphere. The reaction mixture was stirred for a further 1 h at this temperature, before a solution of di-aldehyde **6** in CH_2Cl_2 (100 mL) was added over 20 min *via* pressure-equalising dropping funnel. After stirring for a further 1.5 h at this temperature, the reaction mixture was allowed to come to room temperature over 30 min, and H_2O (200 mL) was added. The layers were separated, and the aqueous layer was extracted with CH_2Cl_2 (3 × 100 mL). The combined organic layers were washed with brine (200 mL), dried with $MgSO_4$, filtered and concentrated under reduced pressure. The crude solid was triturated three times with petroleum spirits, the solvent was removed from the combined extracts under reduced pressure, and the residual oil was purified *via* flash chromatography (SiO_2, petroleum spirits to 5% EtOAc/petroleum spirits) to give 1,3-bis(2,2-dibromovinyl)benzene **7** as a pale yellow solid (4.9 g, 31% over 2 steps).

Spectroscopic data matched those previously reported.[9]

R*f* = 0.50 (5% EtOAc/petroleum spirits);

^{1}H NMR (400 MHz, $CDCl_3$): δ 7.74 – 7.73 (m, 1H), 7.49 – 7.46 (m, 4H), 7.40 – 7.34 (m, 1H) ppm.

Dimethyl 3,3’-(1,3-phenylene)dipropiolate (8)

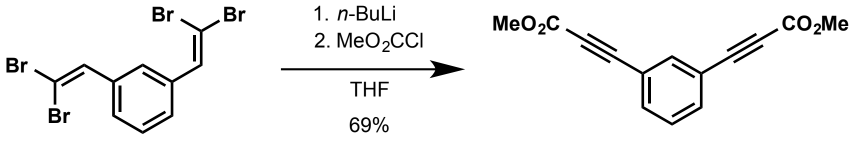

7 **8**

1,3-Benzene-bis(methyl propiolate) **8** was prepared using a modified literature procedure.[5] A solution of tetrabromide **7** (3.5 g, 7.85 mmol, 1.00 mol. equiv.) in THF (40 mL) was added dropwise over 20 min to *n*-BuLi (23.0 mL, 1.4 M in hexanes, 32.2 mmol, 4.1 mol. equiv.) in THF (40 mL) at −78 ºC under a nitrogen atmosphere. The reaction mixture was stirred at this temperature for 1.5 h, before being transferred by cannula into a solution of methyl chloroformate (5.0 mL, 78.5 mmol, 10.0 mol. equiv.) in THF (15 mL) at −78 ºC. After stirring for 15 min at this temperature, the reaction mixture was warmed to room temperature over 1 h, and sat. aq. NH_4Cl (80 mL) was added. The layers were separated and the aqueous layer was extracted with CH_2Cl_2 (3 × 80 mL). The combined organic layers were dried with $MgSO_4$, filtered and concentrated under reduced pressure. The crude product was then purified *via* flash chromatography (SiO_2, petroleum spirits to 20% EtOAc/petroleum spirits) to provide 1,3-benzene-bis(methyl propiolate) **8** as a white crystalline solid (1.32 g, 69%).

Spectroscopic data matched those previously reported.[10]

$\mathbf{R}_f$ = 0.41 (20% EtOAc/petroleum spirits);

^{1}H NMR (400 MHz, $CDCl_3$): δ 7.77 (s, 1H), 7.64 (dd, J = 7.9, 1.3 Hz, 3H), 7.41 (t, J = 7.9 Hz, 2H), 3.85 (s, 6H) ppm.

3,3'-(1,3-phenylene)dipropiolic acid (9)

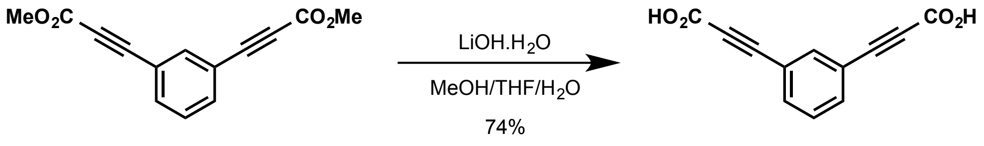

8 9

1,3-Benzene-dipropynoic acid **9** was prepared by adopting and modifying a reported procedure on a structurally related compound.[6] $LiOH.H_2O$ (8.7 mg, 0.206 mmol, 2.50 equiv.) was added to a solution of diester **8** (20 mg, 0.083 mmol, 1.00 equiv.) in 1.5 mL MeOH/THF/H_2O (4:1:1) at room temperature. After stirring at this temp for 20 h, the reaction mixture was acidified (until pH ≤ 2) *via* the dropwise addition of conc. HCl, and extracted with CH_2Cl_2, dried with $MgSO_4$, filtered and concentrated under reduced

pressure. The crude solid was then crsytallised from EtOAc/petroleum spirits to give 1,3-benzene-dipropynoic acid **9** as white powder (13 mg, 74%).

Spectroscopic data matched those previously reported.[7]

^{1}H NMR (400 MHz, DMSO-d_6): δ 7.86 – 7.85 (m, 1H), 7.78 (dd, J = 7.9, 1.6 Hz, 3H), 7.57 (t, J = 7.9 Hz, 2H) ppm.

1,4-Bis(2,2-dibromovinyl)benzene (11)

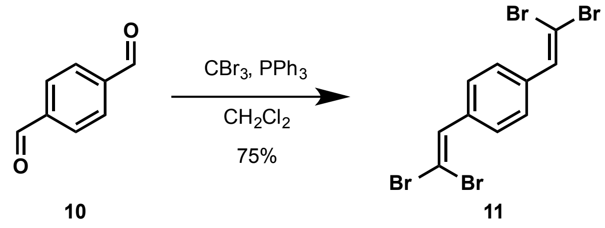

1,4-Bis(2,2-dibromovinyl)benzene **11** was prepared using a modified literature procedure.[4] Triphenylphosphine (36 g, 140 mmol, 4.6 mol. equiv.) was added portionwise over 1 h to a solution of carbon tetrabromide (23 g, 69 mmol, 2.3 mol. equiv.) in CH_2Cl_2 (250 mL) at 0 ºC under a nitrogen atmosphere. The reaction mixture was stirred for a further 1 h at this temperature, before a solution of terephthalaldehyde (**10**) (4.0 g, 30 mmol, 1.0 mol. equiv.) in CH_2Cl_2 (100 mL) was added over 20 min. After stirring for a further 1.5 h at this temperature, the reaction mixture was allowed to come to room temperature over 30 min, and H_2O (100 mL) was added. The layers were separated, and the aqueous layer was extracted with CH_2Cl_2 (3 × 60 mL). The combined organic layers were washed with brine (100 mL), dried with $MgSO_4$, filtered and concentrated under reduced pressure. The crude product was triturated three times with pentane to remove insoluble $Ph_3P{=}O$, and the residual oil was purified *via* flash

chromatography (SiO_2, petroleum spirits to 2.5% EtOAc/petroleum spirits) to give 1,4-bis(2,2-dibromovinyl)benzene **11** as a pale yellow solid (10 g, 75%).

Spectroscopic data matched those previously reported.[11]

R_f = 0.50 (5% EtOAc/petroleum spirits);

^{1}H NMR (400 MHz, $CDCl_3$): δ 7.56 (s, 4H), 7.46 (s, 2H) ppm.

Dimethyl 3,3'-(1,4-phenylene)dipropiolate (12)

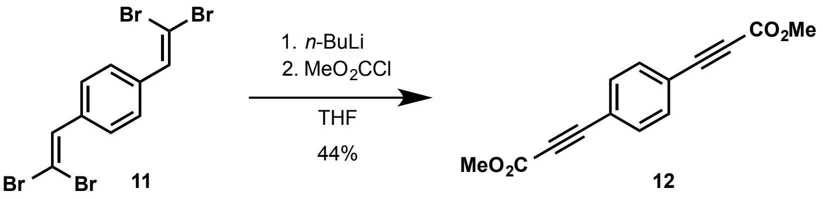

1,4-Benzene-bis(methyl propiolate) **12** was prepared by adopting and modifying the published procedure for the *meta*-isomer.[5] A solution of tetrabromide **11** (2.0 g, 4.5 mmol, 1.0 mol. equiv.) in THF (25 mL) was added dropwise over 20 min to a solution of *n*-BuLi (12 mL, 1.5 M in hexanes, 18 mmol, 4.1 mol. equiv.) in THF (20 mL) at −78 ºC under a nitrogen atmosphere. The reaction mixture was stirred at this temperature for 1.5 h, before being transferred by cannula into a solution of methyl chloroformate (3.5 mL, 45 mmol, 10 mol. equiv.) in THF (15 mL) at −78 ºC. After stirring for 15 min at this temperature, the reaction mixture was warmed to room temperature over 1 h, and sat. aq. NH_4Cl (25 mL) was added. The layers were separated, the aqueous layer was extracted with dichloromethane (3 × 20 mL), and the combined organic layers were dried with $MgSO_4$, filtered and concentrated under reduced pressure. The crude product was then purified *via* recrystallization from EtOAc to generate 1,4-benzene-bis(methyl propiolate) **12** as a white crystalline solid (0.48 g, 44%).

R_f = 0.48 (10% EtOAc/petroleum spirits);

m.p. = 174.7 – 176.1 °C (EtOAc);

^{1}H NMR (400 MHz; $CDCl_3$): δ 7.56 (s, 4H), 3.85 (s, 6H) ppm;

^{13}C NMR (100 MHz; $CDCl_3$): δ 154.1 (C_q), 132.9 (CH), 121.8 (C_q), 84.8 (C_q), 82.5 (C_q), 53.0.1 (C_q), 53.0 (CH_3) ppm;

IR (KBr disc): 3399, 2694, 2226, 1719, 1436 cm^{-1};

HRMS (ESI$^+$): calculated for $[C_{14}H_{10}O_4Na]^+$: 242.0579; found 242.0576.

3,3'-(1,4-phenylene)dipropiolic acid (13)

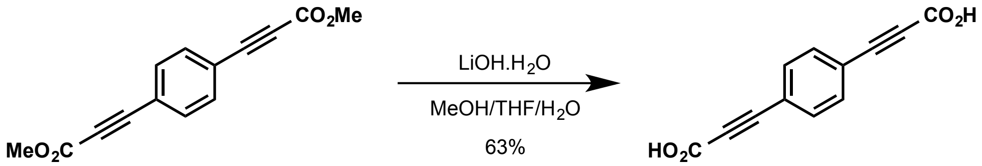

12 13

1,4-Benzene-dipropynoic acid **13** was prepared by adopting and modifying a reported procedure on a structurally related compound.[6] $LiOH.H_2O$ (0.097 g, 2.31 mmol, 2.8 equiv.) was added to a solution of diester **12** (0.20 g, 0.83 mmol, 1.0 equiv.) in 6 mL MeOH/THF/H_2O (4:1:1) at room temperature. After stirring at this temp for 48 h, the reaction mixture was acidified (until pH $\leq$ 2) *via* the dropwise addition of conc. HCl, then extracted with CH_2Cl_2, dried ($MgSO_4$), filtered and concentrated under reduced pressure. The crude solid was then recrystallised from EtOH/EtOAc to give 1,4-benzene-dipropynoic acid **13** as white solid (0.11 g, 63%).

Spectroscopic data matched those previously reported.[7]

^{1}H NMR (400 MHz, DMSO-d_6): δ 7.61 (s, 4H), 3.39 (br s, 2H) ppm.

Mass Spectrometry

The dianions were synthesised by electrospray ionisation of a methanolic solution of 3,3′-(phenylene)dipropiolic acid, basified with aqueous ammonia to aid deprotonation. Mass spectra were acquired using a dual linear quadrupole ion trap mass spectrometer (LTQ Velos Pro, San Jose CA). Parent dianions at *m/z* 106 were isolated and collisionally activated (15% normalised collision energy), yielding the first decarboxylation product at *m/z* 84. The singly-decarboxylated product was subsequently re-isolated and collisionally activated (15% normalised collision energy) to yield the doubly-decarboxylated dianion at *m/z* 62 along with singly-charged CO_2 loss *m/z* 124 and a proton-transfer product (*m/z* 125). This protocol was used for all three isomers.

Ion-molecule experiments were conducted by passing the ion-trap He buffer gas over a small amount of the neutral reagent (for D_2O and C_6H_6). The vapour pressure of the neutral reagent at room temperature was sufficient to seed the He buffer gas and was delivered to the high-pressure cell of the dual ion trap through the unmodified buffer gas inlet and split flow in the mass spectrometer. Reactions with gaseous reagents (D_2 and CD_4) were performed using a pre-made mixture of the deuterated reagents (both purchased from Sigma Aldrich, Castle Hill, NSW) in UHP He (BOC Gases) and delivered into the ion-trap through the ion trap buffer gas He inlet. The proportions of each gas mixture were 1.6% by volume in helium for D_2 and 0.14% by volume in helium for CD_4, yielding estimated number densities of 1.42×10^{12} molecules cm^{-3} and 1.24×10^{11} molecules cm^{-3}, respectively, at the ~2.5 mTorr pressure within the ion trap. The use of D_2O, D_2 and CD_4 was necessitated by the presence of adventitious protonation reagents (such as H_2O and CH_3OH) in the vacuum system.

Theoretical Methods

Standard *ab initio* molecular orbital theory and density functional theory calculations were carried out with Gaussian09.[12] Gas-phase geometries of stationary points were obtained with the BMK/6-31+G(2df,p) procedure.[13] Following each geometry optimisation, harmonic frequency analysis was carried out to confirm the nature of the stationary point as an equilibrium structure (all real frequencies) or a transition structure (one imaginary frequency). To obtain the zero-point vibrational energies (ZPVEs) and

thermal corrections for enthalpies at 298 K (ΔH_{298}) for the fully-optimised structures, we used BMK/6-31+G(2df,p) harmonic vibrational frequencies and appropriate literature scale factors.[14] For each of the *ortho*-, *meta*- and *para*-diethynyl benzenes, we have examined the potential energy surfaces for the mono- and di-anionic species along the path that connects the geometries of the two ions. This is accomplished using structures obtained through a linear combination of the optimised structures for the mono- and di-anions (see Figure 4 of the main manuscript). Improved single-point energies were evaluated using the G4(MP2)-6X procedure for all structures.[15] All relative energies are given in kJ mol^{-1}.

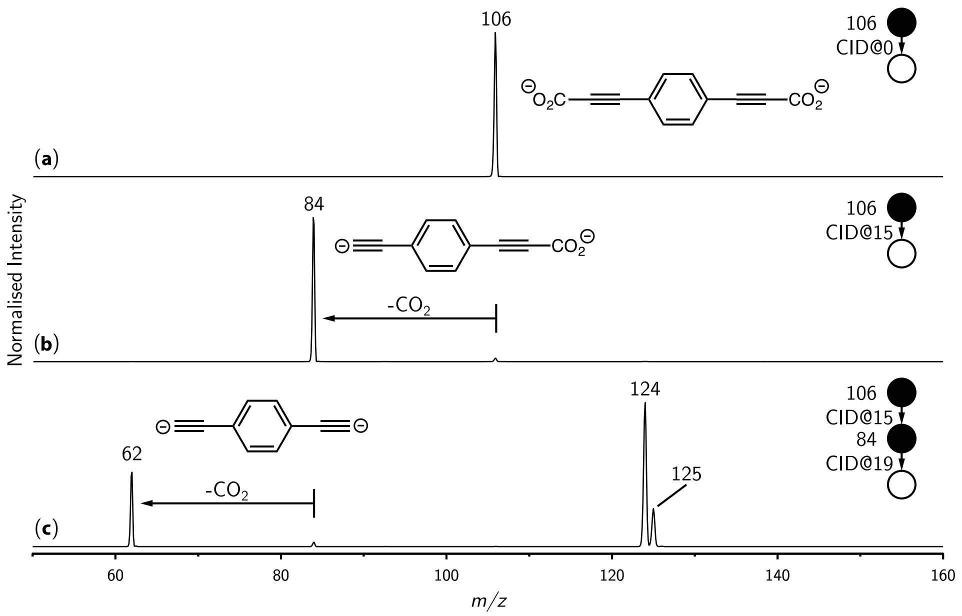

Supporting Figure 1: Mass spectra illustrating the synthesis of the *para*-DEB dianion base, analogous to Figure 1 of the manuscript. The mass-isolated dicarboxylate anion at *m/z* 106 (a) is observed to decarboxylate under CID to yield *m/z* 84 (b). Subsequent isolation and activation of this *m/z* 84 ion yields a second decarboxylation product at *m/z* 62 and associated reaction products (c).

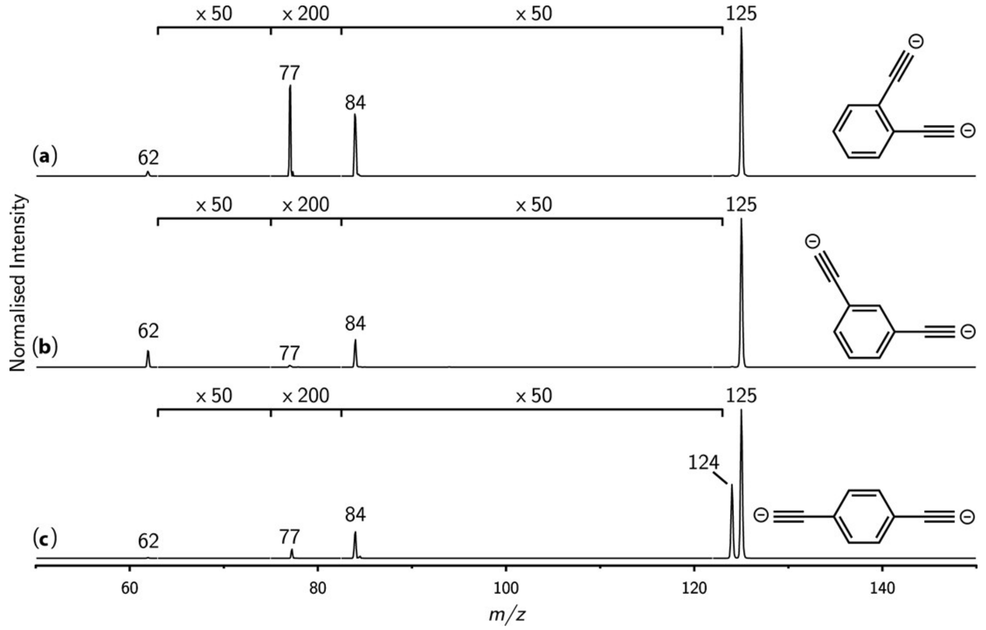

Supporting Figure 2: Comparison of the reactivity of (a) *ortho*- (b) *meta*- and (c) *para*-DEB^{2-} (*m/z* 62) isomers towards benzene (C_6H_6). Reaction time is 100 ms. Note the region around *m/z* 77 has been enlarged by a factor of 200 in each spectrum.

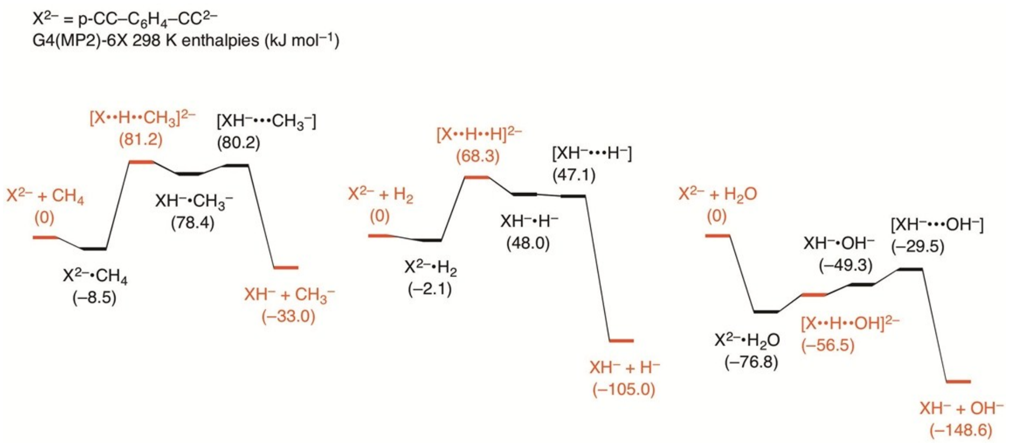

Supporting Figure 3: Calculated potential energy diagrams for proton transfer to *para*-DEB^{2-} from CH_4, H_2 and H_2O.

Supporting Table 1: Barriers to electron loss in kJ mol^{-1} determined from the VDE and AEA using Equation 1 (RCB[Marcus]) and from a rectilinear projection of the dianion and monoanion geometries as described in the text and shown in Figure 4 of the manuscript (RCB[Analytic]).

	**RCB [Marcus] (kJ mol^{-1})**	**RCB [Analytic] (kJ mol^{-1})**
**[*ortho*-DEB]$^{2-}$**	11.1	16.5
**[*meta*-DEB]$^{2-}$**	52.7	51.2
**[*para*-DEB]$^{2-}$**	1.9	1.8

Supporting Table 2: Selected Neutral Reagents, their Conjugate Bases, G4(MP2)-6X computed proton affinities and the experimentally reported Proton Affinities of the Conjugate Bases.

**Neutral Reagent**	**Conjugate Base**	**Calculated Proton Affinity (kJ mol^{-1})**	**Experimental Proton Affinity (kJ mol^{-1})**	**Experimental Reference**
CH_4	CH_3^-	1747.7	1743.6±2.9	16, 17
			1749±15	17, 18
			1742.2±0.8	19
NH_3	NH_2^-	1688.6	1688.0±1.2	17, 20
			1688.5±3.3	17, 21
D_2	D^-		1678.663±0.042	17, 22
H_2	H^-	1675.7 *	1675.5	17, 22
C_6H_6	$C_6H_5^-$	1675.7	1678.7±2.1	17, 23, 24
D_2O	DO^-		1636.61±0.25	17, 25
H_2O	HO^-	1632.1	1633.141±0.042	17, 25

*Calculated at the CCSD/AV6Z level as the G4(MP2)-6X method does not include the diffuse functions required to describe the hydride anion.

Supporting Table 3: Calculated values for the Proton Affinity (PA), Vertical Detachment Energy (VDE), Adiabatic Electron Affinity (AEA) and Repulsive Coulomb Barrier height (RCB) for the methylene-linked diacetylide dianions [G4(MP2)-6X, kJ mol^{-1}]

**Dianion**	**PA (kJ mol^{-1})**	**VDE (kJ mol^{-1})**	**AEA (kJ mol^{-1})**	**RCB (kJ mol^{-1})**
$[C_2(CH_2)_4C_2]^{2-}$	1775.4	97.6	86.1	206.8
$[C_2(CH_2)_3C_2]^{2-}$	1806.1	60.5	42.1	49.8
$[C_2(CH_2)_2C_2]^{2-}$	1836.4	22.2	1.4	5.9
$[C_2(CH_2)_1C_2]^{2-}$	1888.1	–28.7	–77.1	4.2
$[C_2(CH_2)_0C_2]^{2-}$	1937.8	–161.6	–57.4	–62.6

Supporting Table 4: Optimized BMK/6-31+G(2df,p) structures (Å) and vibrationless G4(MP2)-6X energies (hartrees) for relevant species

***p*-DEB^{2-}**
G4(MP2)-6X = -382.79687

C	0.000000	1.197304	0.697954
C	0.000000	1.197304	-0.697954
C	0.000000	0.000000	-1.455087
C	0.000000	-1.197304	-0.697954
C	0.000000	-1.197304	0.697954
C	0.000000	0.000000	1.455087
H	0.000000	2.142257	1.239346
H	0.000000	2.142257	-1.239346
H	0.000000	-2.142257	-1.239346
H	0.000000	-2.142257	1.239346
C	0.000000	0.000000	-2.896121
C	0.000000	0.000000	-4.144366
C	0.000000	0.000000	2.896121
C	0.000000	0.000000	4.144366

***p*-DEB^{-}**
G4(MP2)-6X = -382.79896

C	0.000000	1.224053	0.686804
C	0.000000	1.224053	-0.686804
C	0.000000	0.000000	-1.434542
C	0.000000	-1.224053	-0.686804
C	0.000000	-1.224053	0.686804
C	0.000000	0.000000	1.434542
H	0.000000	2.157164	1.244231
H	0.000000	2.157164	-1.244231
H	0.000000	-2.157164	-1.244231
H	0.000000	-2.157164	1.244231
C	0.000000	0.000000	-2.829585
C	0.000000	0.000000	-4.093529
C	0.000000	0.000000	2.829585
C	0.000000	0.000000	4.093529

***p*-DEBH^{-}**
G4(MP2)-6X = -383.48441

C	0.000000	1.208843	0.764904
C	0.000000	1.210194	-0.623299
C	0.000000	0.000000	-1.350137
C	0.000000	-1.210194	-0.623299
C	0.000000	-1.208843	0.764904
C	0.000000	0.000000	1.515829
H	0.000000	2.147779	1.312445
H	0.000000	2.151396	-1.169192
H	0.000000	-2.151396	-1.169192
H	0.000000	-2.147779	1.312445
C	0.000000	0.000000	-2.781633
C	0.000000	0.000000	-3.990795
H	0.000000	0.000000	-5.055377
C	0.000000	0.000000	2.934007
C	0.000000	0.000000	4.184330

***m*-DEB^{2-}**
G4(MP2)-6X = -382.79206

C	1.244860	0.113879	0.000000
C	0.000000	0.787276	0.000000
C	-1.245131	0.113735	0.000000
C	-1.217637	-1.304336	0.000000
C	0.000084	-1.990585	0.000000
C	1.217566	-1.304451	0.000000
H	-0.000330	1.874034	0.000000
H	-2.163386	-1.841795	0.000000
H	-0.000069	-3.082274	0.000000
H	2.163420	-1.841716	0.000000
C	-2.504449	0.816628	0.000000
C	-3.615890	1.383782	0.000000
C	2.504511	0.816300	0.000000
C	3.616147	1.383065	0.000000

***m*-DEB^{-}**
G4(MP2)-6X = -382.77803

C	1.228117	0.158690	0.000000
C	0.000000	0.847127	0.000000
C	-1.227978	0.158504	0.000000
C	-1.203804	-1.280294	0.000000
C	-0.000078	-1.981717	0.000000
C	1.203584	-1.280083	0.000000
H	-0.000100	1.932238	0.000000
H	-2.158343	-1.799915	0.000000
H	0.000132	-3.069137	0.000000
H	2.158082	-1.799857	0.000000
C	-2.486491	0.792707	0.000000
C	-3.644807	1.290715	0.000000
C	2.486648	0.792849	0.000000
C	3.644849	1.290947	0.000000

***m*-DEBH^{-}**
G4(MP2)-6X = -383.48205

C	-1.298158	0.187981	0.000000
C	0.000000	0.758072	0.000000
C	1.154602	-0.037777	0.000000
C	1.054389	-1.443408	0.000000
C	-0.218761	-2.022008	0.000000
C	-1.367334	-1.231040	0.000000
H	0.091687	1.840305	0.000000
H	1.954328	-2.051404	0.000000
H	-0.313204	-3.106659	0.000000
H	-2.352645	-1.690154	0.000000
C	2.454715	0.581137	0.000000
C	3.547556	1.092825	0.000000
C	-2.470167	0.994685	0.000000
C	-3.504705	1.695040	0.000000
H	4.507002	1.554881	0.000000

***o*-DEB^{2-}**
G4(MP2)-6X = -382.76781

C	0.000000	-0.760330	0.000000
C	-0.458590	0.607244	0.000000
C	0.528999	1.629995	0.000000
C	1.901622	1.378190	0.000000
C	2.347759	0.047161	0.000000
C	1.404424	-0.981029	0.000000
H	0.163957	2.655265	0.000000

H	2.609126	2.208262	0.000000
H	3.412771	-0.187788	0.000000
H	1.731296	-2.019003	0.000000
C	-0.840566	-1.923742	0.000000
C	-1.414113	-3.032725	0.000000
C	-1.830815	1.028074	0.000000
C	-2.958245	1.564373	0.000000

***o*-DEB^{-}**
G4(MP2)-6X = -382.78344

C	0.000000	-0.760486	0.000000
C	-0.434691	0.625925	0.000000
C	0.575218	1.648053	0.000000
C	1.916554	1.348116	0.000000
C	2.342748	-0.011496	0.000000
C	1.412545	-1.023058	0.000000
H	0.231497	2.678703	0.000000
H	2.653613	2.148540	0.000000
H	3.404841	-0.247421	0.000000
H	1.719249	-2.065249	0.000000
C	-0.872580	-1.847868	0.000000
C	-1.557652	-2.911297	0.000000
C	-1.772385	1.018958	0.000000
C	-2.944624	1.494058	0.000000

***o*-DEBH^{-}**
G4(MP2)-6X = -383.47989

C	0.000000	-0.677383	0.000000
C	0.300018	0.722075	0.000000
C	-0.819468	1.601661	0.000000
C	-2.129898	1.141159	0.000000
C	-2.403342	-0.238545	0.000000
C	-1.333377	-1.130255	0.000000
H	-0.609043	2.667943	0.000000
H	-2.950959	1.856694	0.000000
H	-3.426814	-0.605255	0.000000
H	-1.511689	-2.203516	0.000000
C	1.052397	-1.652465	0.000000
C	1.912534	-2.498308	0.000000
H	2.721386	-3.190354	0.000000
C	1.619932	1.237529	0.000000
C	2.764058	1.740280	0.000000

LiO^{-}
G4(MP2)-6X = -82.59555013

O	0.000000	0.000000	0.475278
Li	0.000000	0.000000	-1.267407

LiOH
G4(MP2)-6X = -83.28207689

Li	0.000000	0.000000	-1.265815
O	0.000000	0.000000	0.316657
H	0.000000	0.000000	1.264189

CH_4
G4(MP2)-6X = -40.47484

C	0.000000	0.000000	0.000000
H	0.632158	0.632158	0.632158
H	-0.632158	-0.632158	0.632158
H	-0.632158	0.632158	-0.632158
H	0.632158	-0.632158	-0.632158

H_2
G4(MP2)-6X = -1.18178

H	0.000000	0.000000	0.371049
H	0.000000	0.000000	-0.371049

H_2O
G4(MP2)-6X = -76.38151

O	0.000000	0.000000	0.115305
H	0.000000	0.765519	-0.461222
H	0.000000	-0.765519	-0.461222

***p*-DEB^{2-}•CH_4**
G4(MP2)-6X = -423.27714

C	-1.753434	1.197514	-0.000023
C	-0.357518	1.197375	0.001104
C	0.398302	-0.000137	0.001715
C	-0.357688	-1.197545	0.001104
C	-1.753604	-1.197490	-0.000026
C	-2.510313	0.000065	-0.000649
H	-2.294802	2.142376	-0.000448
H	0.183999	2.142200	0.001528
H	0.183700	-2.142444	0.001524
H	-2.295101	-2.142277	-0.000458
C	1.839682	-0.000242	0.002862
C	3.086705	-0.000370	0.004009
C	-3.950944	0.000170	-0.001764
C	-5.199057	0.000229	-0.002684
H	5.651167	0.000163	-0.000306
C	6.755707	0.000273	-0.003489
H	7.131173	-0.000301	1.028125
H	7.126325	0.894831	-0.520941
H	7.126515	-0.893599	-0.521974

***p*-DEB^{2-}••H^{+}••CH_3^{-}**
G4(MP2)-6X = -423.23503

C	-1.640762	1.200746	0.000194
C	-0.246417	1.201050	-0.000222
C	0.495777	0.000006	-0.000428
C	-0.246411	-1.201042	-0.000194
C	-1.640755	-1.200746	0.000222
C	-2.394457	-0.000002	0.000446
H	-2.183892	2.143625	0.000352
H	0.297243	2.144012	-0.000380
H	0.297255	-2.144000	-0.000331
H	-2.183880	-2.143629	0.000401
C	1.937903	0.000010	-0.000756
C	3.162451	0.000011	-0.000652
C	-3.829362	-0.000006	0.000891
C	-5.077585	-0.000010	0.001285
H	4.416208	0.000007	-0.001204
C	6.109667	-0.000010	-0.000587
H	6.523304	0.000045	1.031516
H	6.526746	0.892793	-0.515833
H	6.526714	-0.892892	-0.515723

_p_-DEBH$^-$•CH_3^-

G4(MP2)-6X = -423.23998

C	-1.688518	1.202593	0.000306
C	-0.295309	1.203341	-0.000454
C	0.440821	0.000011	-0.000890
C	-0.295294	-1.203327	-0.000531
C	-1.688503	-1.202597	0.000230
C	-2.440996	-0.000006	0.000692
H	-2.232506	2.144541	0.000629
H	0.249322	2.145456	-0.000718
H	0.249348	-2.145435	-0.000854
H	-2.232479	-2.144551	0.000494
C	1.881268	0.000021	-0.001636
C	3.094860	0.000054	-0.002022
C	-3.872589	-0.000015	0.001450
C	-5.121047	-0.000024	0.002095
H	4.208499	0.000050	-0.002822
C	6.490359	-0.000033	-0.000033
H	6.901601	-0.000076	1.034178
H	6.913000	0.892771	-0.513053
H	6.912900	-0.892863	-0.513090

_p_-DEB^{2-}•H_2

G4(MP2)-6X = -383.98225

C	0.483953	1.197427	-0.000020
C	-0.912002	1.197468	0.000008
C	-1.668851	0.000006	0.000005
C	-0.912019	-1.197466	0.000003
C	0.483937	-1.197444	-0.000025
C	1.240074	-0.000014	-0.000056
H	1.025383	2.142306	-0.000017
H	-1.453391	2.142335	0.000031
H	-1.453420	-2.142325	0.000022
H	1.025352	-2.142332	-0.000027
C	-3.109580	0.000015	0.000055
C	-4.357701	0.000020	0.000097
C	2.681368	-0.000021	-0.000060
C	3.928532	-0.000034	-0.000072
H	6.475678	0.000170	0.000177
H	7.234144	0.000101	0.000204

_p_-DEB^{2-}••H^+••H^-

G4(MP2)-6X = -383.98224

C	0.000000	1.197487	-0.484042
C	0.000000	1.197520	0.911862
C	0.000000	0.000000	1.668700
C	0.000000	-1.197520	0.911862
C	0.000000	-1.197487	-0.484042
C	0.000000	0.000000	-1.240169
H	0.000000	2.142263	-1.025657
H	0.000000	2.142274	1.453449
H	0.000000	-2.142274	1.453449
H	0.000000	-2.142263	-1.025657
C	0.000000	0.000000	3.109406
C	0.000000	0.000000	4.357505
C	0.000000	0.000000	-2.681441
C	0.000000	0.000000	-3.928583
H	0.000000	0.000000	-6.471683
H	0.000000	0.000000	-7.230248

_p_-DEBH$^-$•H^-

G4(MP2)-6X = -383.96344

C	-0.511782	1.192298	-0.000011
C	0.872331	1.192777	-0.000002
C	1.611988	0.000002	0.000000
C	0.872335	-1.192774	-0.000014
C	-0.511779	-1.192300	-0.000022
C	-1.237697	-0.000002	-0.000023
H	-1.046467	2.126516	-0.000004
H	1.406798	2.125956	0.000007
H	1.406805	-2.125952	-0.000013
H	-1.046459	-2.126520	-0.000023
C	3.047831	0.000003	0.000008
C	4.279633	0.000001	0.000000
C	-2.681643	-0.000002	-0.000002
C	-3.878894	-0.000012	0.000052
H	-4.959465	0.000005	0.000194
H	-6.935140	0.000052	-0.000076

_p_-DEB^{2-}•H_2O

G4(MP2)-6X = -459.20923

C	-1.592027	-1.198769	-0.025210
C	-0.198069	-1.162988	-0.081616
C	0.523489	0.053935	-0.089760
C	-0.257425	1.232402	-0.038486
C	-1.651491	1.196709	0.017588
C	-2.376831	-0.020367	0.027005
H	-2.109570	-2.156619	-0.020607
H	0.366908	-2.092948	-0.120480
H	0.260287	2.190295	-0.043239
H	-2.216247	2.126667	0.055624
C	1.964034	0.088348	-0.144155
C	3.204063	0.115050	-0.191069
C	-3.814277	-0.056212	0.084735
C	-5.061145	-0.085554	0.133335
H	5.047651	0.045689	-0.085542
O	6.028587	-0.074030	0.133539
H	5.980356	-0.496168	0.991720

_p_-DEB^{2-}••H^+••OH^-

G4(MP2)-6X = -459.19649

C	-1.580723	-1.189833	-0.003176
C	-0.195007	-1.189169	-0.025950
C	0.538905	0.000207	-0.037033
C	-0.195236	1.189439	-0.025700
C	-1.580953	1.189828	-0.002933
C	-2.323253	-0.000075	0.009831
H	-2.113005	-2.124886	0.005257
H	0.337798	-2.124609	-0.035127
H	0.337389	2.124983	-0.034672
H	-2.113416	2.124777	0.005694
C	1.985580	0.000341	-0.056666
C	3.196222	0.000439	-0.069697
C	-3.763511	-0.000220	0.035460
C	-4.995227	-0.000348	0.058443
H	4.462608	0.000367	-0.054565
O	5.811215	0.000134	-0.009898
H	6.078114	-0.005358	0.897116

***p*-DEBH⁻•OH⁻**

G4(MP2)-6X = -459.20923

C	-1.651455	-1.196710	0.017581
C	-0.257399	-1.232373	-0.038508
C	0.523499	-0.053885	-0.089799
C	-0.198078	1.163025	-0.081633
C	-1.592024	1.198775	-0.025218
C	-2.376817	0.020352	0.026987
H	-2.216207	-2.126669	0.055628
H	0.260357	-2.190239	-0.043246
H	0.366894	2.092987	-0.120491
H	-2.109614	2.156597	-0.020601
C	1.964040	-0.088310	-0.144177
C	3.204071	-0.115120	-0.191074
C	-3.814254	0.056198	0.084754
C	-5.061125	0.085496	0.133391
H	5.047607	-0.045673	-0.085632
O	6.028507	0.074032	0.133587
H	5.980159	0.496056	0.991820

$C_2(CH_2)_0C_2^{2-}$

G4(MP2)-6X = -151.96160

C	0.000000	0.000000	1.956697
C	0.000000	0.000000	0.701463
C	0.000000	0.000000	-0.701463
C	0.000000	0.000000	-1.956697

$C_2(CH_2)_0C_2^{-}$

G4(MP2)-6X = -151.98344

C	0.000000	0.000000	1.930233
C	0.000000	0.000000	0.696642
C	0.000000	0.000000	-0.696642
C	0.000000	0.000000	-1.930233

$C_2(CH_2)_0C_2H^{-}$

G4(MP2)-6X = -152.70833

C	0.000000	0.000000	2.048057
C	0.000000	0.000000	0.797097
C	0.000000	0.000000	-0.574523
C	0.000000	0.000000	-1.794418
H	0.000000	0.000000	-2.857283

$C_2(CH_2)_1C_2^{2-}$

G4(MP2)-6X = -191.22399

C	-1.304668	-0.045938	0.000000
C	-2.453524	-0.528186	0.000000
C	0.000000	0.691519	0.000000
H	-0.000245	1.368303	0.873995
H	-0.000245	1.368303	-0.873995
C	1.304766	-0.045524	0.000000
C	2.453507	-0.527971	0.000000

$C_2(CH_2)_1C_2^{-}$

G4(MP2)-6X = -191.25337

C	-1.056978	0.030723	0.000000
C	-1.974518	-0.838414	0.000000
C	0.000000	1.053180	0.000000
H	0.000128	1.686356	0.896870
H	0.000128	1.686356	-0.896870
C	1.057024	0.030786	0.000000
C	1.974430	-0.838394	0.000000

$C_2(CH_2)_1C_2H^{-}$

G4(MP2)-6X = -191.95234

C	-1.154303	-0.098954	0.000000
C	-2.114147	-0.828181	0.000000
H	-2.930875	-1.510781	0.000000
C	0.000000	0.828277	0.000000
H	-0.120459	1.484672	0.878316
H	-0.120459	1.484672	-0.878316
C	1.328164	0.175755	0.000000
C	2.468918	-0.319992	0.000000

$C_2(CH_2)_2C_2^{2-}$

G4(MP2)-6X = -230.51866

C	-1.350613	1.404045	0.000000
C	-2.472159	1.947496	0.000000
C	0.000000	0.779811	0.000000
H	0.582312	1.104479	0.877362
H	0.582312	1.104479	-0.877362
C	-0.000181	-0.779877	0.000000
H	-0.582293	-1.104593	0.877438
H	-0.582293	-1.104593	-0.877438
C	1.350724	-1.404002	0.000000
C	2.472221	-1.947435	0.000000

$C_2(CH_2)_2C_2^{-}$

G4(MP2)-6X = -230.51812

C	-1.872399	0.158014	0.000237
C	-3.071207	-0.235711	-0.000726
C	-0.507230	0.635548	0.000338
H	-0.259314	1.222211	0.891522
H	-0.259297	1.222578	-0.890596
C	0.507187	-0.635565	0.000328
H	0.259581	-1.222142	0.891590
H	0.259501	-1.222485	-0.890674
C	1.872526	-0.158101	0.000254
C	3.071044	0.235789	-0.000737

$C_2(CH_2)_2C_2H^{-}$

G4(MP2)-6X = -231.22758

C	-1.995699	0.171728	0.000000
C	-3.171884	-0.236816	0.000000
C	-0.591221	0.618033	0.000000
H	-0.354446	1.236834	0.880801
H	-0.354447	1.236834	-0.880801
C	0.389420	-0.593306	0.000000
H	0.178286	-1.213161	0.880639
H	0.178286	-1.213161	-0.880639
C	1.806484	-0.200368	0.000000
C	2.958864	0.154959	0.000000
H	3.976535	0.467273	0.000001

$C_2(CH_2)_3C_2^{2-}$

G4(MP2)-6X = -269.79765

C	-2.580118	-0.104149	0.000000
C	-3.693691	-0.662279	-0.000096
C	-1.281739	0.617285	0.000068

H	-1.222404	1.286122	0.878234
H	-1.222331	1.286093	-0.878115
C	-0.000065	-0.255188	0.000118
H	-0.000241	-0.911022	0.881294
H	-0.000300	-0.911202	-0.880927
C	1.281748	0.617030	-0.000040
H	1.222520	1.285920	0.878144
H	1.222237	1.285938	-0.878202
C	2.580151	-0.104191	-0.000208
C	3.693800	-0.662150	0.000087

$C_2(CH_2)_3C_2^-$
G4(MP2)-6X = -269.78160

C	-2.468568	-0.119051	0.000000
C	-3.512227	-0.829890	0.000000
C	-1.279985	0.720770	0.000000
H	-1.236865	1.367813	0.889133
H	-1.236865	1.367813	-0.889133
C	0.000000	-0.181007	0.000000
H	0.000069	-0.824297	0.885782
H	0.000069	-0.824297	-0.885782
C	1.279951	0.720835	0.000000
H	1.236659	1.367858	0.889146
H	1.236659	1.367858	-0.889146
C	2.468717	-0.118855	0.000000
C	3.512158	-0.829926	0.000000

$C_2(CH_2)_3C_2H^-$
G4(MP2)-6X = -270.49499

C	-2.608075	-0.136663	-0.000054
C	-3.641570	-0.831065	-0.000005
C	-1.378203	0.677800	-0.000054
H	-1.340707	1.345393	0.880670
H	-1.340566	1.345144	-0.880955
C	-0.085448	-0.170678	0.000132
H	-0.077948	-0.822580	0.882533
H	-0.077849	-0.822872	-0.882058
C	1.176476	0.725358	0.000026
H	1.157088	1.382532	0.881953
H	1.157019	1.382436	-0.881974
C	2.437785	-0.028006	0.000017
C	3.460311	-0.664840	-0.000073
H	4.355323	-1.241491	-0.000102

$C_2(CH_2)_4C_2^{2-}$
G4(MP2)-6X = -309.07877

C	3.223120	-0.075188	-0.000012
C	4.370367	0.409335	0.000059
C	1.866855	-0.678639	-0.000028
H	1.738132	-1.335644	0.878271
H	1.738153	-1.335667	-0.878313
C	0.694088	0.334078	-0.000047
H	0.797338	0.986695	0.880426
H	0.797291	0.986600	-0.880595
C	-0.694086	-0.334060	0.000028
H	-0.797293	-0.986575	0.880579
H	-0.797337	-0.986680	-0.880440
C	-1.866859	0.678652	-0.000004
H	-1.738142	1.335700	0.878264
H	-1.738140	1.335640	-0.878317
C	-3.223128	0.075190	0.000013
C	-4.370356	-0.409380	0.000013

$C_2(CH_2)_4C_2^-$
G4(MP2)-6X = -309.04599

C	3.137341	-0.082867	-0.000121
C	4.281849	0.448153	-0.000292
C	1.831577	-0.744226	0.000104
H	1.706115	-1.382312	0.886984
H	1.705884	-1.382453	-0.886640
C	0.695852	0.326443	0.000152
H	0.816298	0.966959	0.883733
H	0.816215	0.966930	-0.883464
C	-0.695846	-0.326418	0.000220
H	-0.816236	-0.966863	0.883861
H	-0.816235	-0.966996	-0.883325
C	-1.831569	0.744212	0.000122
H	-1.706096	1.382363	0.886961
H	-1.705878	1.382415	-0.886646
C	-3.137362	0.082859	-0.000093
C	-4.281854	-0.448162	-0.000335

$C_2(CH_2)_4C_2H^-$
G4(MP2)-6X = -309.76474

C	3.269413	-0.100749	-0.000282
C	4.386062	0.449112	-0.000572
C	1.931525	-0.725898	0.000174
H	1.795943	-1.379767	0.880585
H	1.795516	-1.380099	-0.879924
C	0.782342	0.309482	0.000244
H	0.898068	0.957184	0.881434
H	0.897978	0.957188	-0.880955
C	-0.612625	-0.334640	0.000281
H	-0.733296	-0.978984	0.882974
H	-0.733283	-0.979112	-0.882319
C	-1.735281	0.728879	0.000154
H	-1.623817	1.376385	0.881282
H	-1.623570	1.376474	-0.880879
C	-3.088071	0.153434	-0.000090
C	-4.185998	-0.341531	-0.000215
H	-5.157744	-0.777807	-0.000356

^1H and ^{13}C NMR Spectra

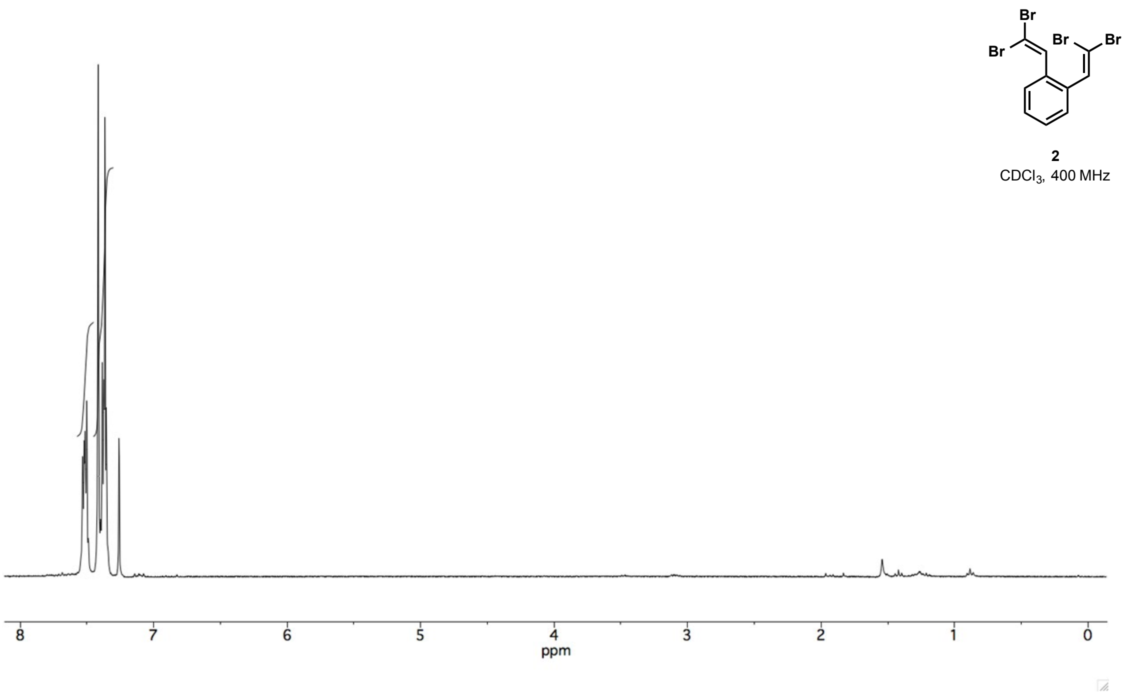

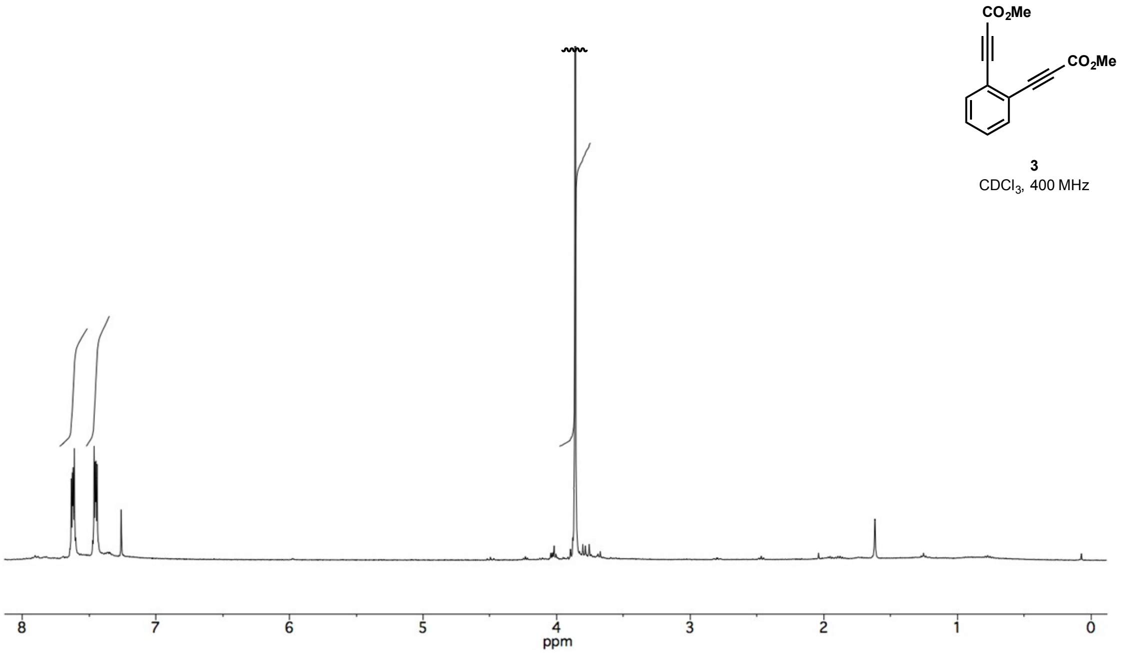

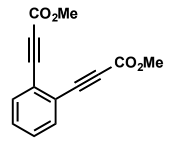

3
$CDCl_3$, 100 MHz

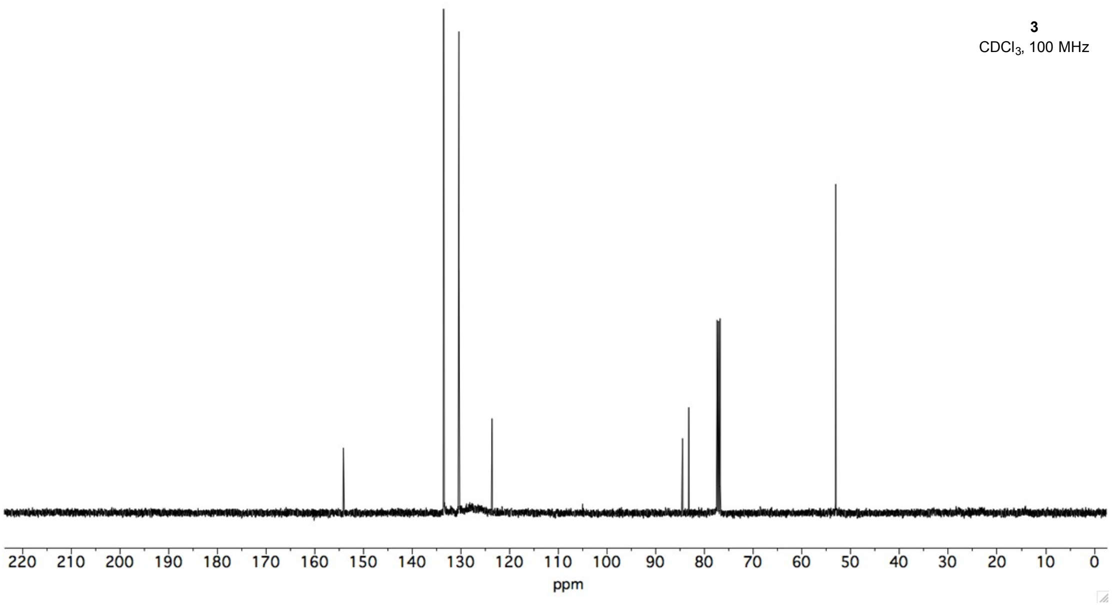

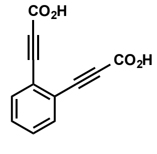

4
CD_3CN, 400 MHz

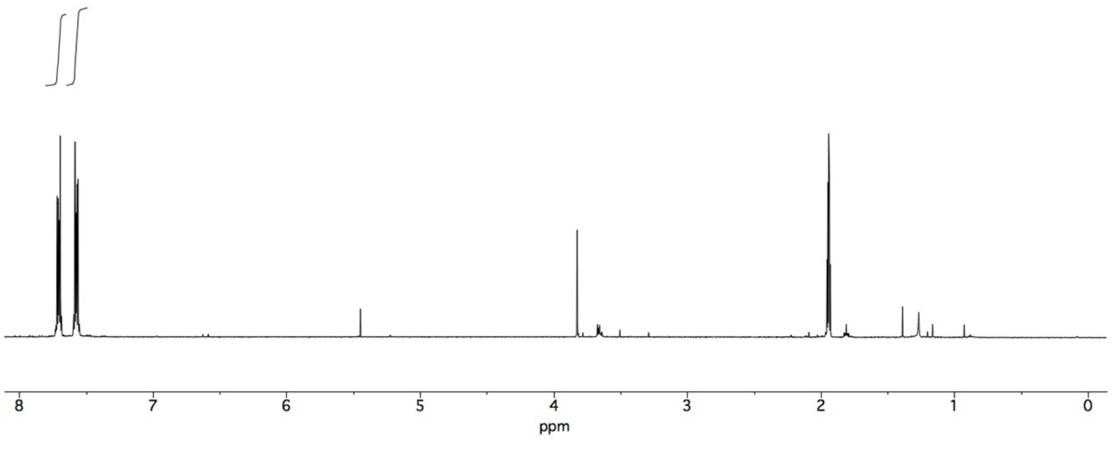

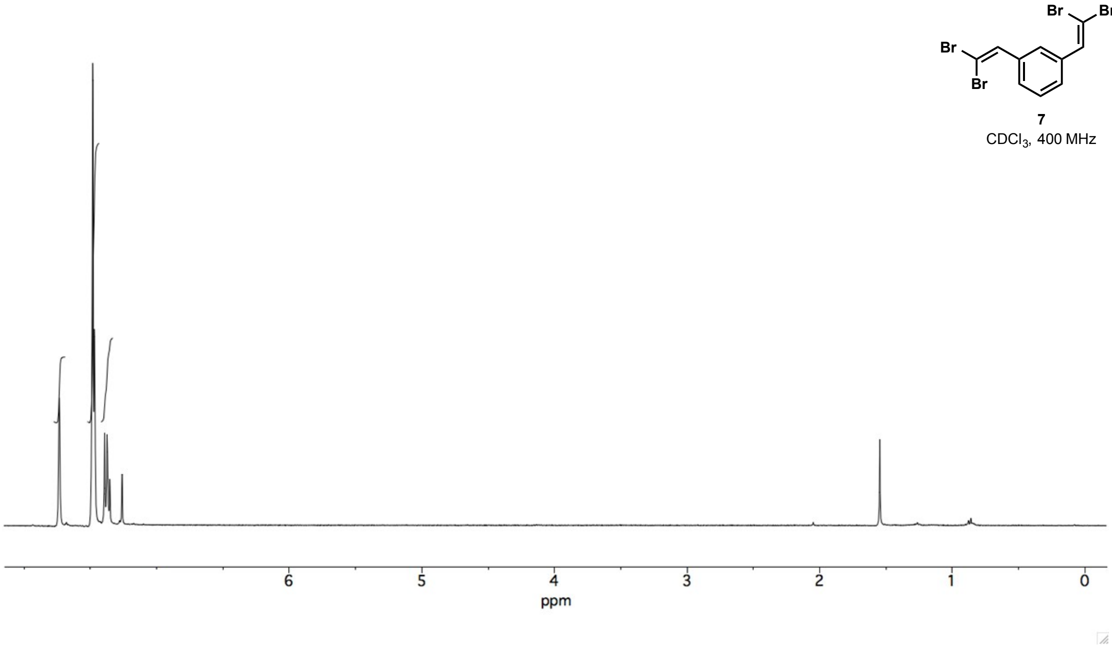

7
$CDCl_3$, 400 MHz

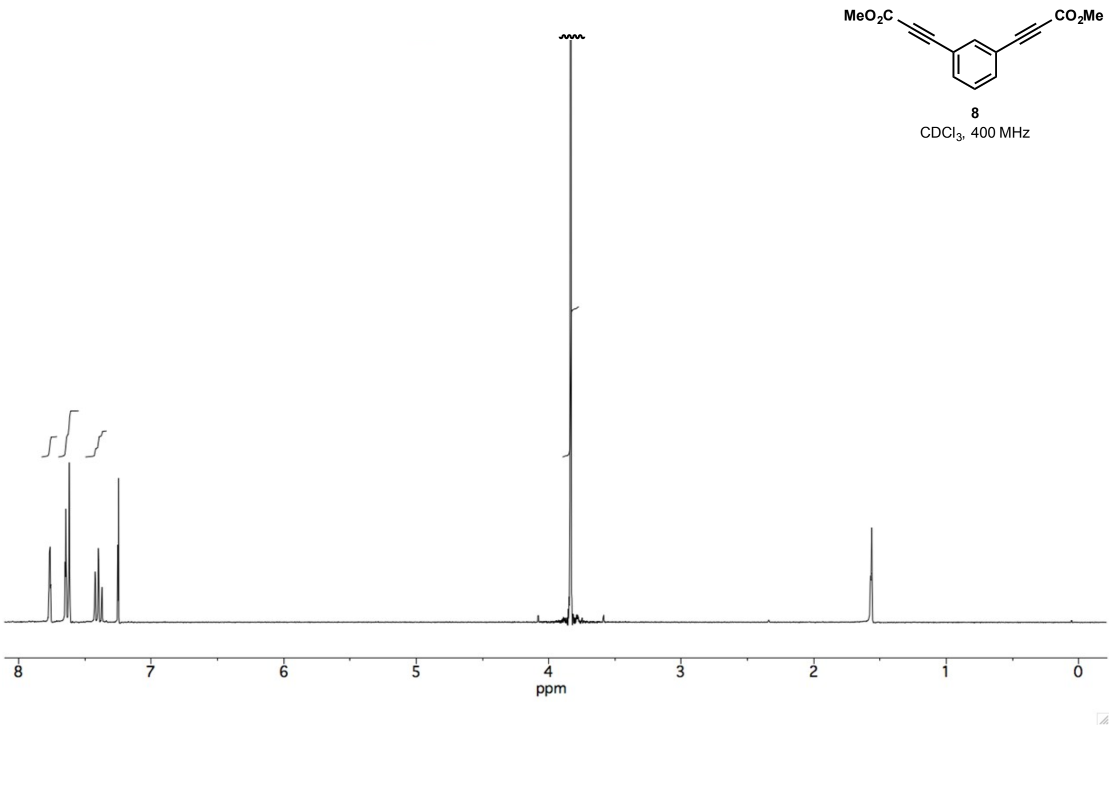

8
$CDCl_3$, 400 MHz

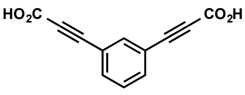

9
$CDCl_3$, 400 MHz

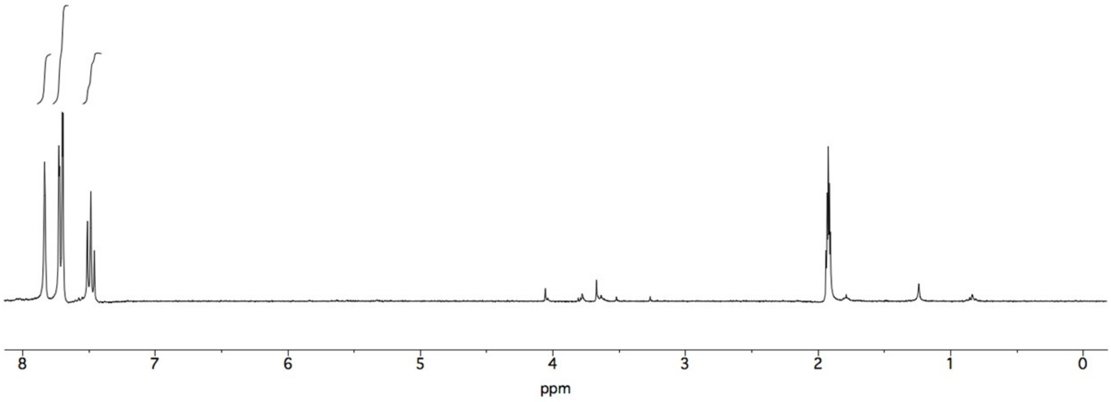

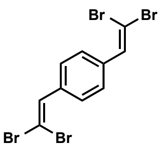

11
$CDCl_3$, 400 MHz

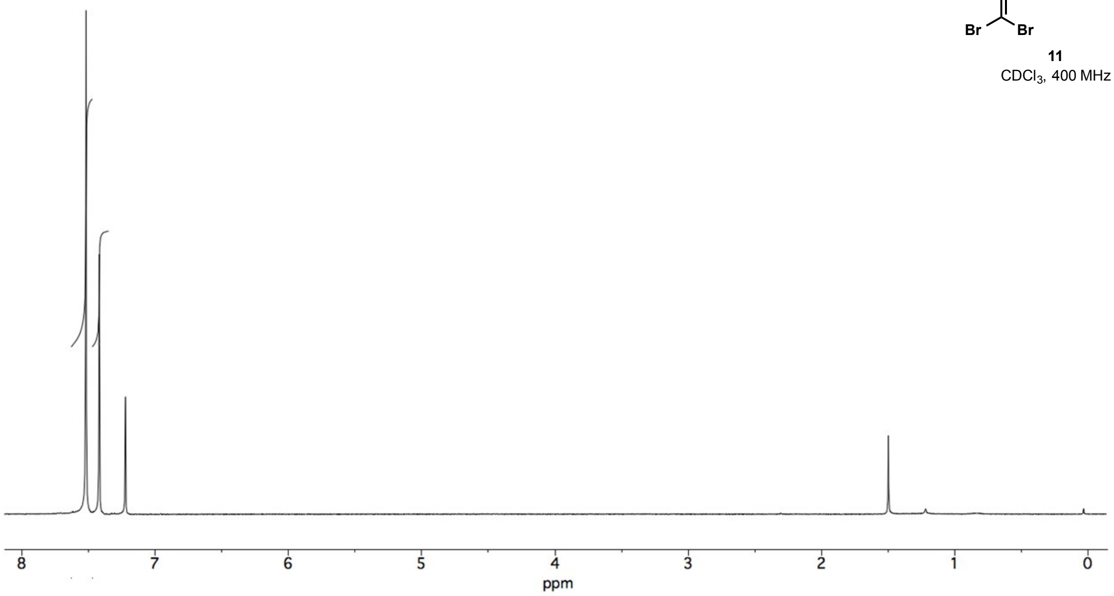

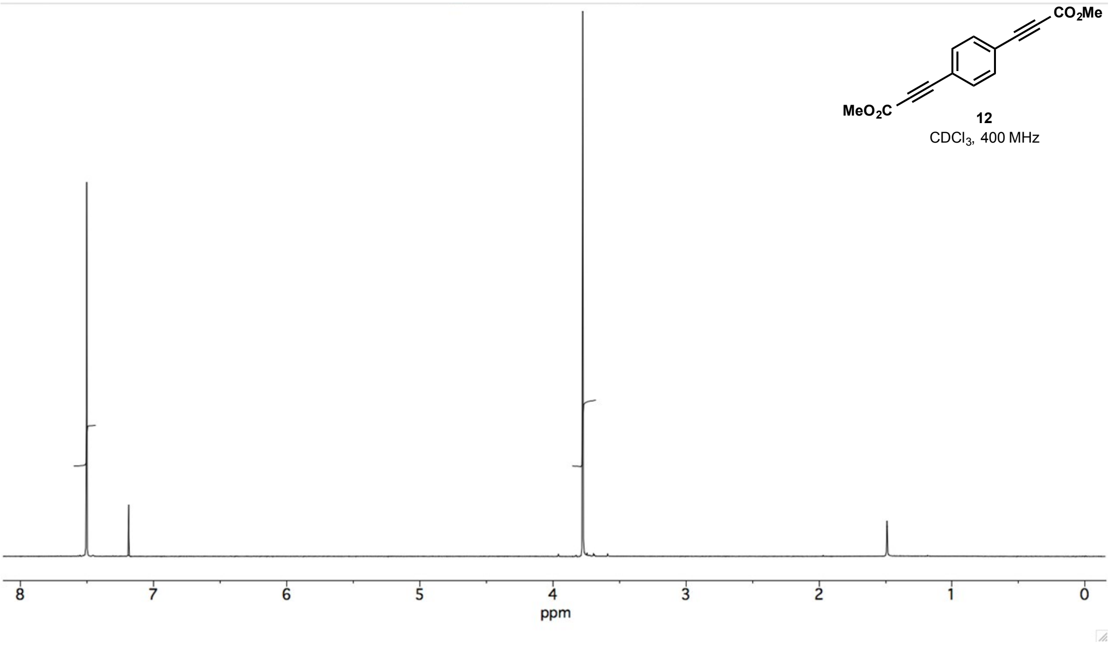
CO2Me
MeO2C
12
CDCl3, 400 MHz
8 7 6 5 4 3 2 1 0
ppm

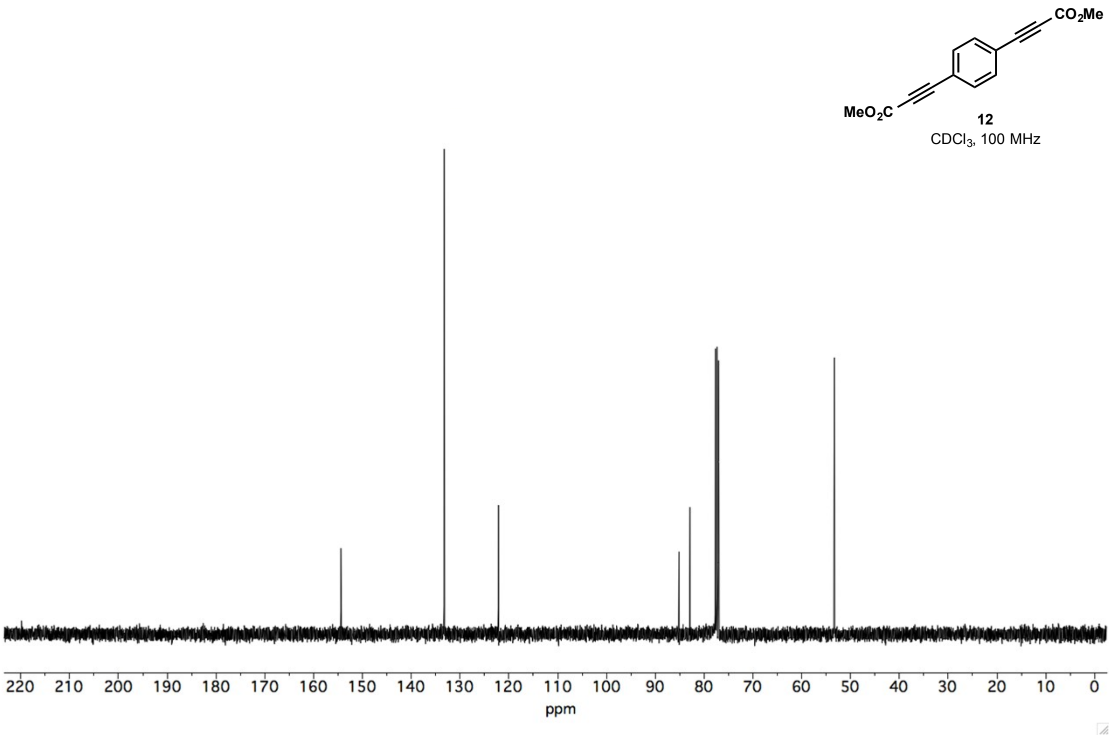
CO2Me
MeO2C
12
CDCl3, 100 MHz
220 210 200 190 180 170 160 150 140 130 120 110 100 90 80 70 60 50 40 30 20 10 0
ppm

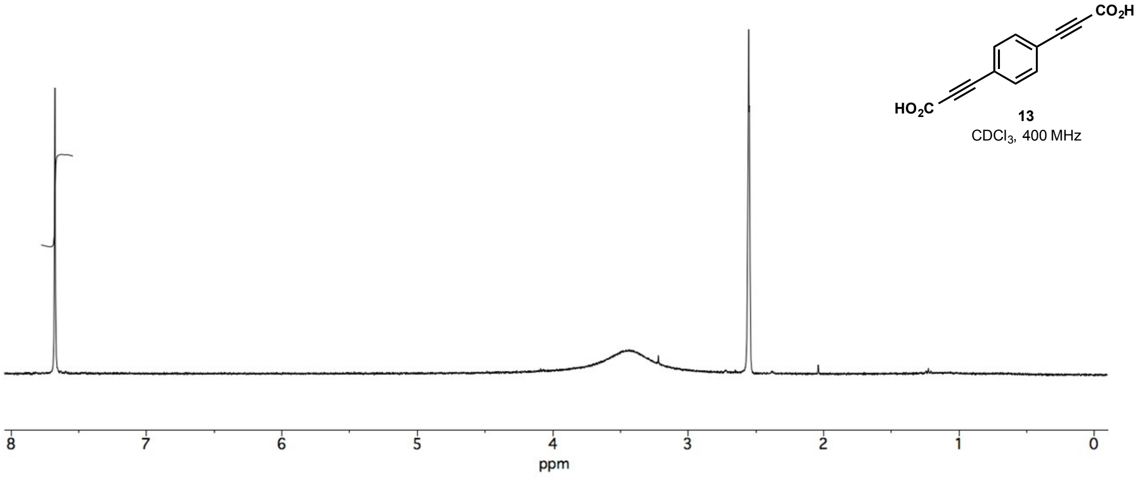

CO2H
HO2C
13
CDCl3, 400 MHz
8
7
6
5
4
3
2
1
0
ppm

Supporting References

1. A. B. Pangborn, M. A. Giardello, R. H. Grubbs, R. K. Rosen and F. J. Timmers, *Organometallics*, 1996, **15**, 1518-1520.
2. W. L. F. Armarego and C. Chai, *Purification of laboratory chemicals*, Butterworth-Heinemann, Oxford, 5th edn., 2009.
3. H. S. Lin and L. A. Paquette, *Synth. Comm.*, 1994, **24**, 2503-2506.
4. B. Sahu, R. Muruganantham and I. N. N. Namboothiri, *Eur. J. Org. Chem.*, 2007, **2007**, 2477-2489.
5. Y. Yamamoto and N. Kirai, *Org. Lett.*, 2008, **10**, 5513-5516.
6. M. Ohashi, K. Gamo, Y. Tanaka, M. Waki, Y. Beniyama, K. Matsuno, J. Wada, M. Tenta, J. Eguchi, M. Makishima, N. Matsuura, T. Oyama and H. Miyachi, *Eur. J. Med. Chem.*, 2015, **90**, 53-67.
7. R. Saravanakumar, B. Varghese and S. Sankararaman, *CrystEngComm*, 2009, **11**, 337-346.
8. M. Hirano, S. Yakabe, H. Chikamori, J. H. Clark and T. Morimoto, *J. Chem. Res.*, 1998, 770-771.
9. M. L. N. Rao, D. N. Jadhav and P. Dasgupta, *Org. Lett.*, 2010, **12**, 2048-2051.
10. U. Kumar and T. X. Neenan, *Macromolecules*, 1995, **28**, 124-130.
11. G. T. Hwang, H. S. Son, J. K. Ku and B. H. Kim, *J. Am. Chem. Soc.*, 2003, **125**, 11241-11248.
12. M. J. Frisch, G. W. Trucks, H. B. Schlegel, G. E. Scuseria, M. A. Robb, J. R. Cheeseman, G. Scalmani, V. Barone, B. Mennucci, G. A. Petersson, H. Nakatsuji, M. Caricato, X. Li, H. P. Hratchian, A. F. Izmaylov, J. Bloino, G. Zheng, J. L. Sonnenberg, M. Hada, M. Ehara, K. Toyota, R. Fukuda, J. Hasegawa, M. Ishida, T. Nakajima, Y. Honda, O. Kitao, H. Nakai, T. Vreven, J. A. Montgomery Jr., J. E. Peralta, F. Ogliaro, M. J. Bearpark, J. Heyd, E. N. Brothers, K. N. Kudin, V. N. Staroverov, R. Kobayashi, J. Normand, K. Raghavachari, A. P. Rendell, J. C. Burant, S. S. Iyengar, J. Tomasi, M. Cossi, N. Rega, N. J. Millam, M. Klene, J. E. Knox, J. B. Cross, V. Bakken, C. Adamo, J. Jaramillo, R. Gomperts, R. E. Stratmann, O. Yazyev, A. J. Austin, R. Cammi, C. Pomelli, J. W. Ochterski, R. L. Martin, K. Morokuma, V. G. Zakrzewski, G. A. Voth, P. Salvador, J. J. Dannenberg, S. Dapprich, A. D. Daniels, Ö. Farkas, J. B. Foresman, J. V. Ortiz, J. Cioslowski and D. J. Fox, GAUSSIAN09 Revision C01, 2009.
13. A. D. Boese and J. M. L. Martin, *J. Chem. Phys.*, 2004, **121**, 3405-3416.
14. J. P. Merrick, D. Moran and L. Radom, *J. Phys. Chem. A*, 2007, **111**, 11683-11700.
15. B. Chan, J. Deng and L. Radom, *J. Chem. Theory Comput.*, 2011, **7**, 112-120.
16. G. B. Ellison, P. C. Engelking and W. C. Lineberger, *J. Am. Chem. Soc.*, 1978, **100**, 2556-2558.
17. J. E. Bartmess, in *NIST Chemistry WebBook, NIST Standard Reference Database Number 69*, eds. P. J. Linstrom and W. G. Mallard, National Institute of Standards and Technology, Gaithersburg MD, 20899, retrieved April 13, 2016.
18. S. T. Graul and R. R. Squires, *J. Am. Chem. Soc.*, 1990, **112**, 2517.
19. A. M. Oliveira, Y.-J. Lu, J. H. Lehman, P. B. Changala, J. H. Baraban, J. F. Stanton and W. C. Lineberger, *J. Am. Chem. Soc.*, 2015, **137**, 12939-12945.
20. C. T. Wickham-Jones, K. M. Ervin, G. B. Ellison and W. C. Lineberger, *J. Chem. Phys.*, 1989, **91**, 2762-2763.

21. G. J. MacKay, R. S. Hemsworth and D. K. Bohme, *Can. J. Chem.*, 1976, **54**, 1624-1642.
22. K. R. Lykke, K. K. Murray and W. C. Lineberger, *Phys. Rev. A*, 1991, **43**, 6104-6107.
23. G. E. Davico, V. M. Bierbaum, C. H. DePuy, G. B. Ellison and R. R. Squires, *J. Am. Chem. Soc.*, 1995, **117**, 2590-2599.
24. K. M. Ervin and V. F. DeTuro, *J. Phys. Chem. A*, 2002, **106**, 9947-9956.
25. P. A. Schulz, R. D. Mead, P. L. Jones and W. C. Lineberger, *J. Chem. Phys.*, 1982, **77**, 1153-1165.
